# Functionalization of Metal-Enriched *Pleurotus* Biomass with γ-Poly-Glutamic Acid for Cytoprotective and Antimicrobial Wound Care

**DOI:** 10.3390/ph19101627

**Published:** 2026-10-14

**Authors:** Agata Krakowska, Alicja Matyjewicz, Iwona Skiba-Kurek, Rafał Twaróg, Mateusz Marzec, Żaneta Binert-Kusztal, Paulina Koczurkiewicz-Adamczyk, Bożena Muszyńska, Tomasz Skalski

**Affiliations:** 1 Department of Analytical Chemistry and Biochemistry, Faculty of Materials Science and Ceramics, AGH University of Krakow, Al. Mickiewicza 30, 30-059 Kraków, Poland; amatyjewicz@agh.edu.pl; 2 Department of Inorganic Chemistry and Pharmaceutical Analytics, Faculty of Pharmacy, Jagiellonian University Medical College, 9 Medyczna Street, 30-688 Kraków, Poland; zaneta.binert@uj.edu.pl; 3 Department of Microbiology, University Hospital, Marii Orwid 11 Street, 30-688 Kraków, Poland; iskiba@su.krakow.pl; 4 Department of Glass Technology and Amorphous Coatings, Faculty of Materials Science and Ceramics, AGH University of Krakow, Al. A. Mickiewicza 30, 30-059 Kraków, Poland; rtwarog@agh.edu.pl; 5 Department of Surface Nanoengineering and Biomaterials, Academic Centre for Materials and Nanotechnology, AGH University of Krakow, Al. A. Mickiewicza 30, 30-059 Kraków, Poland; marzecm@agh.edu.pl; 6 Department of Pharmaceutical Biochemistry, Faculty of Pharmacy, Jagiellonian University Medical College, 9 Medyczna Street, 30-688 Kraków, Poland; paulina.koczurkiewicz@uj.edu.pl; 7 Department of Medical Plant and Mushroom Biotechnology, Faculty of Pharmacy, Jagiellonian University Medical College, 9 Medyczna Street, 30-688 Kraków, Poland; bozena.muszynska@uj.edu.pl; 8 Biotechnology Centre, Silesian University of Technology, Krzywoustego 8 Street, 44-100 Gliwice, Poland; tomasz.skalski@polsl.pl

**Keywords:** fungal biomass, γ-poly(glutamic acid), biofortification, wound dressing, cytocompatibility, antimicrobial activity, surface modification, biomaterial

## Abstract

**Objectives**: Chronic wound dressings require active components that support moisture management, biocompatibility, and local bioburden control. Therefore, in this study, freeze-dried biomass of *Pleurotus ostreatus* (Po) and *Pleurotus djamor* (Pdj) was cultivated in vitro under mineral-enriched conditions (calcium, magnesium, zinc) and subsequently modified with poly(γ-glutamic acid) (γ-PGA) to develop a bioactive fungal matrix intended as a functional additive for future wound-care biomaterials. **Methods**: The elemental content of the biomass was determined using atomic absorption spectroscopy (AAS), and the resulting biomaterial underwent physicochemical analyses—including scanning electron microscopy, X-ray diffraction (XRD), wettability measurements, differential scanning calorimetry (DSC), and thermogravimetric analysis (TG)—as well as biological performance assessments using a disk diffusion assay and cytotoxicity analysis. **Results**: Mineral enrichment enhanced *Pleurotus* spp. biomass production and metal accumulation, while γ-PGA coating altered surface properties and cytocompatibility. Zinc supplementation significantly promoted biomass formation in both *Pleurotus* species, while metal accumulation depended on the specific element and fortification regime. γ-PGA modification reduced the accessible BET surface area and formed a denser polymer-associated layer, confirmed by X-ray photoelectron spectroscopy. While the unmodified Po biomass exhibited the strongest in vitro antibacterial activity against *S. aureus* and *P. aeruginosa*, it also displayed dose-dependent cytotoxicity toward HaCaT keratinocytes at high concentrations. The addition of γ-PGA moderately reduced the in vitro bacterial inhibition zones but provided a critical cytoprotective effect, mitigating the cytotoxicity of the concentrated fungal extracts and rescuing HaCaT-cell viability to meet ISO 10993-5 safety thresholds. **Conclusions**: These findings demonstrate that γ-PGA-modified, metal-enriched *Pleurotus* biomass exhibits a distinct in vitro trade-off between absolute antimicrobial potency and cellular safety, supporting its further investigation as a biocompatible ingredient for multifunctional wound-dressing systems.

## 1. Introduction

Wound healing is a highly regulated physiological process that includes hemostasis, inflammation, proliferation, and remodeling [1,2]. Chronic wounds arise when this sequence is disrupted, leading to a prolonged inflammatory state driven by persistent infections, biofilm formation, excessive reactive oxygen species (ROS), or insufficient cellular and molecular signals required for tissue repair. Consequently, such wounds fail to progress toward resolution and remain affected by ongoing inflammation and tissue damage [3,4]. Such wounds require therapeutic approaches addressing multiple interdependent factors simultaneously, as encapsulated in the M.O.I.S.T. concept: Moisture balance, Oxygen balance, Infection control, Supporting strategies, and Tissue management—a framework developed to standardize and guide the local therapy of chronic wounds [5].

In recent years, there has been a growing interest in biomaterials of natural origin in medical applications, particularly in tissue engineering and the design of modern wound dressings [6,7]. Multifunctional dressings, which, in addition to protecting the wound from the external environment, also have antimicrobial properties, support tissue regeneration, and control wound exudate, are being increasingly developed [8]. Among the dressing technologies developed to meet these needs, hydrogels have emerged as one of the most versatile platforms. Their biocompatibility, moisture retention, swelling capacity, and oxygen permeability have made them a promising choice for wound repair, as they address the key disadvantages of conventional dry dressings, which fail to support active healing and can aggravate pain through adhesion to the wound bed [9,10,11,12].

Despite their numerous advantages, conventional hydrogel dressings exhibit limited mechanical stability and insufficient bioactivity [13,14]. Therefore, increasing attention has been directed toward the incorporation of functional additives that can enhance both the physicochemical and biological performance of hydrogel-based wound dressings [10,14,15].

Fungal biomass is a natural additive that can be incorporated into hydrogel matrices because of its rich content of bioactive compounds [16,17,18]. Edible mushrooms contain polysaccharides, phenolic compounds, terpenoids, proteins, and other metabolites that exhibit antioxidant, antimicrobial, immunomodulatory, and tissue-regenerative effects [19,20,21]. Furthermore, the main components of the fungal cell wall, chitin, β-glucans, and glycoproteins, contribute to its biocompatibility and potential wound-healing properties [22,23,24]. Additionally, fungi exhibit a remarkable capacity to absorb and accumulate various compounds from their growth substrate. This feature enables the enrichment of fungal biomass with selected bioactive molecules, trace elements, or other functional agents, thereby providing additional opportunities for tailoring the biological performance of wound dressing materials [25,26,27].

One such compound is poly(γ-glutamic acid) (γ-PGA), a naturally occurring, biodegradable, and biocompatible polypeptide produced mainly by *Bacillus* spp. [28,29]. This compound exhibits exceptional water-retention capacity and swelling ability, which are desirable characteristics for wound dressing applications [30]. In addition, γ-PGA may promote cell adhesion and proliferation, tissue regeneration, exhibit anti-inflammatory and antibacterial properties, and can be used as a drug carrier [28,30,31]. As a result, the incorporation of γ-PGA into fungal biomass may represent a promising strategy for combining the bioactivity of mushroom-derived compounds with the favorable physicochemical characteristics of this biopolymer.

However, an important gap remains in the development of fungal biomass-based wound-care systems. Previous studies have mainly considered fungal biomass as a naturally bioactive additive or have focused separately on mineral biofortification, polymeric hydrogel design, or the biological activity of mushroom-derived compounds [6,7,8,9,10,11,12,13,14,15,16,17,18,25,26,27]. Such approaches do not fully address the need to simultaneously control the biochemical composition of the biomass, its interfacial properties, and its safety toward wound-relevant cells. Native fungal matrices may provide antimicrobial and antioxidant activity [16,17,18,19,20,21,22,23,24], but their complex mixture of metabolites can also produce concentration-dependent effects on mammalian cells. Conversely, conventional hydrogel systems offer favorable hydration, swelling, and exudate-management properties but often require functional additives to improve their mechanical and biological performance [9,10,11,12,13,14,15]. In addition, although mineral biofortification can tailor the elemental composition of *Pleurotus* biomass [25,26,27], it has received limited attention in combination with subsequent surface functionalization designed to modulate hydration-related properties, bioactive-compound availability, and cytocompatibility.

Here, we hypothesized that a sequential design strategy mineral biofortification of *Pleurotus* biomass during in vitro cultivation followed by non-covalent γ-PGA coating- could generate a more functionally balanced bioactive matrix than unmodified fungal biomass alone. The fungal component was selected as a renewable, chitin- and β-glucan-rich carrier of intrinsic bioactive metabolites and as a biosorbent platform capable of accumulating selected mineral elements [16,17,18,19,20,21,22,23,24,25,26,27]. Calcium, magnesium, and zinc were introduced to tailor the mineral profile of the biomass [25,26,27], whereas γ-PGA was applied to provide a hydrophilic, carboxyl-rich polymeric interface with high water-binding capacity and potential cytoprotective function [28,29,30,31]. Thus, the intended role of γ-PGA was not simply to add another bioactive constituent, but to modulate the balance between the antimicrobial activity of the fungal matrix and its compatibility with host cells. The novelty of this work lies in combining cultivation-stage Ca/Mg/Zn enrichment with post-cultivation γ-PGA functionalization of *Pleurotus* biomass and in evaluating how these modifications affect mineral accumulation, surface and thermal properties, wettability, antimicrobial performance, and keratinocyte cytocompatibility.

Therefore, rather than evaluating a fully formulated medical device, this study focuses strictly on developing and characterizing a novel active biomaterial ingredient. Specifically, we investigated the potential of edible mushroom biomass coated with poly(γ-glutamic acid) to serve as a multifunctional, bioactive additive for future hydrogel-based wound dressings. To this end, two species from the genus *Pleurotus* spp. were used to form a γ-PGA-modified fungal matrix intended for subsequent incorporation into hydrogel systems. The standalone functionalized biomass was then subjected to comprehensive characterization to evaluate its baseline physicochemical properties and biological performance prior to future hydrogel formulation. The physicochemical properties of the developed materials were evaluated through wettability measurements, scanning electron microscopy (SEM), X-ray diffraction (XRD), differential scanning calorimetry (DSC), and thermogravimetric analysis (TG), providing insight into their surface characteristics, morphology, structural organization, and thermal stability. The biological performance was assessed by microbiological testing against *Enterococcus fecalis* (*E. fecalis*), *Staphylococcus aureus* (*S. aureus*), and *Pseudomonas aeruginosa* (*P. aueruginosa*), while cytotoxicity studies on skin cells were conducted to evaluate their biocompatibility and potential suitability for wound-healing applications.

## 2. Results and Discussion

### 2.1. Biomass Growth

**Figure 1:**
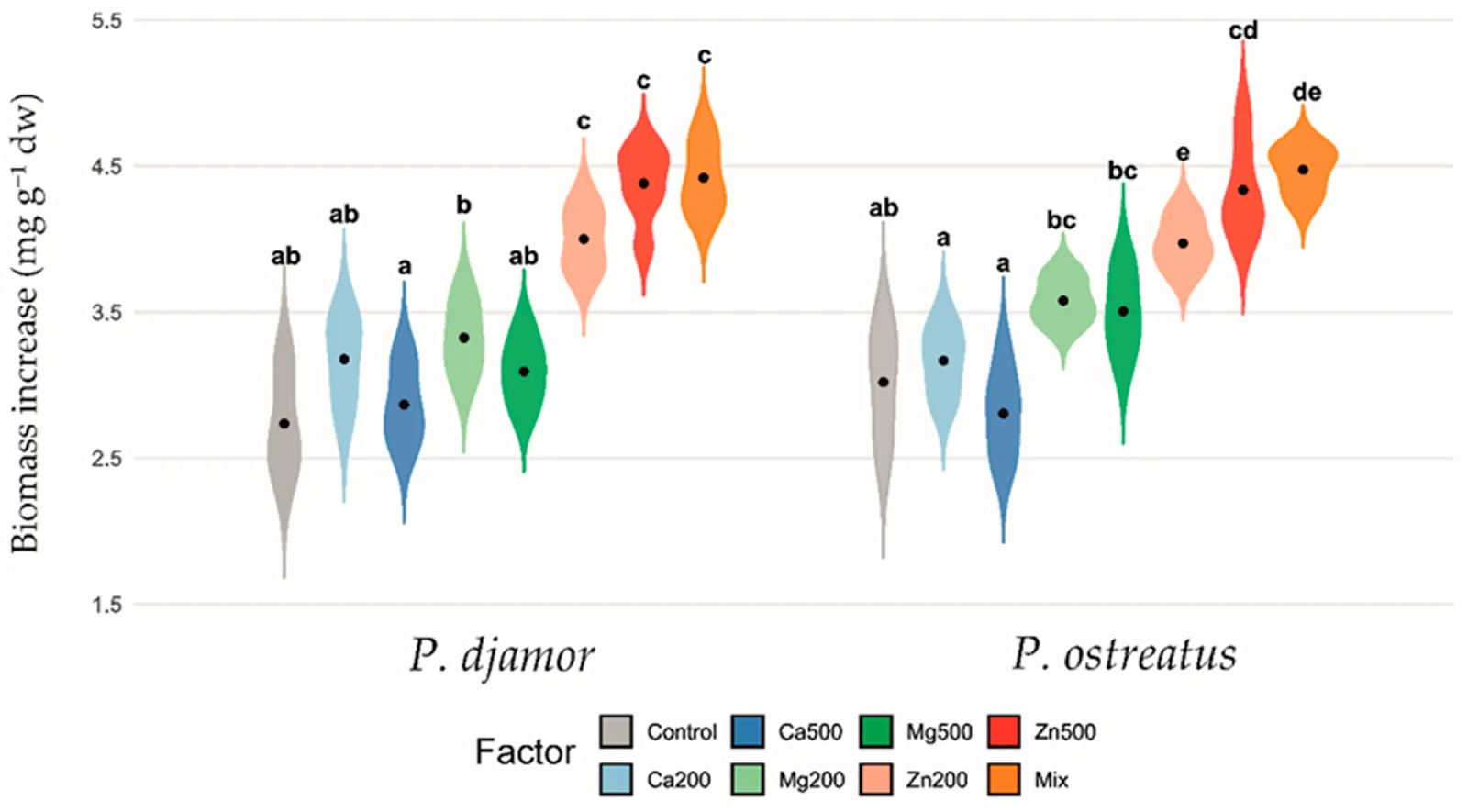
Influence of metal fortification on the biomass increase of *Pleurotus djamor* (Pdj) and *Pleurotus ostreatus* (Po) during cultivation in liquid Oddoux medium. The violin plots illustrate the distribution of biomass growth (mg g^−1^ dw) across different treatments: an unfortified control, independent additions of calcium (Ca), magnesium (Mg), and zinc (Zn) at doses of 200 mg and 500 mg, and a multielement mixture (Mix). Black dots within the violins represent the model-estimated marginal means. Different lowercase letters above the distributions indicate statistically significant differences (*p* < 0.05) among the treatments within each fungal strain, as determined by a generalized linear mixed model (GLMM) and subsequent post-hoc analysis.

The application of generalized linear mixed models (GLMM) indicated distinct differences in biomass accumulation across the various experimental treatments. Based on the mean values presented in Figure 1, treatments fortified with zinc—specifically at doses of 200 and 500, as well as the multi-element mix—yielded the highest biomass levels in both the Pdj and Po fungal strains when compared to the unfortified control and other individual mineral treatments such as calcium or magnesium. Furthermore, biomass production increased with zinc concentration in both *Pleurotus* strains, with the Zn500 treatment generally resulting in higher biomass yields than Zn200. This response suggests that zinc availability influenced mycelial growth under the applied culture conditions. As zinc is required for the activity of numerous fungal enzymes and for the maintenance of cellular functions, the stronger response observed at the higher supplementation level may reflect improved metabolic performance of the cultures. These findings indicate that zinc concentration should be considered when optimizing *Pleurotus* cultivation conditions for biomass production.

The pronounced dose-dependent stimulation of *Pleurotus* biomass by zinc supplementation underscores the indispensable role of this micronutrient as a catalytic and structural cofactor in fungal physiology [32,33]. While calcium and magnesium yielded only moderate growth effects, the significant superiority of zinc—both individually at high doses (500 mg) and within the multielement mix—suggests that it acts as a primary rate-limiting nutrient in standard liquid media [26]. In fungi, zinc is recognized as a strong intracellular antioxidant and an essential component of numerous metalloenzymes, acting as a direct cofactor for superoxide dismutase and enzymes responsible for the metabolism of proteins, carbohydrates, and nucleic acids. The robust growth observed at the 500 mg dose further indicates that *P. djamor* and *P. ostreatus* not only possess high zinc tolerance but actively assimilate it to stabilize the molecular structure of subcellular organelles and cell membranes, thereby accelerating cellular proliferation. Furthermore, external literature confirms that targeted zinc supplementation effectively increases the overall protein content and free-radical scavenging activity specifically in *P. ostreatus* mycelium [34,35,36,37]. Consequently, optimizing zinc availability serves a crucial dual purpose: it acts as a highly effective biostimulant to maximize mycelial yield during cultivation, while simultaneously biofortifying the resulting matrix for subsequent functional applications [38,39].

### 2.2. Changes in Biomass Accumulation

**Figure 2:**
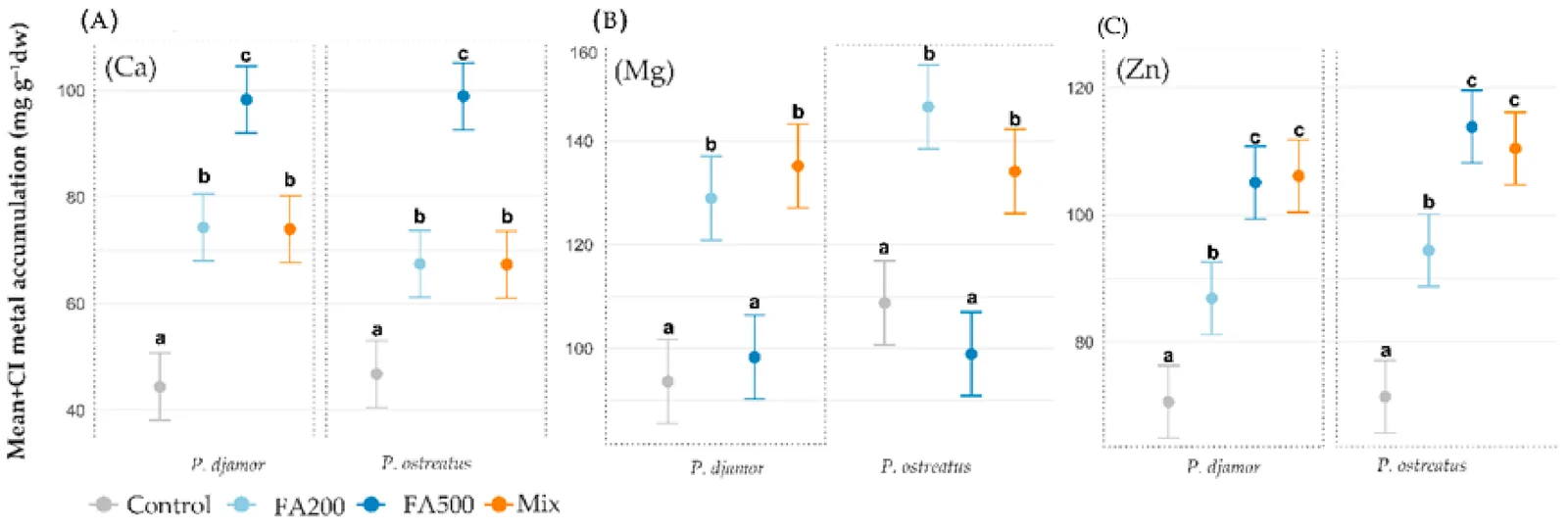
Generalized linear mixed model (GLMM) estimated means of metal accumulation in *Pleurotus djamor* (Pdj) and *Pleurotus ostreatus* (Po) strains under various fortification treatments. The panels display the accumulation of (**A**) calcium, (**B**) magnesium, and (**C**) zinc. Central data points represent the model-estimated marginal means, while the vertical error bars indicate the 95% confidence intervals (CI). Different lowercase letters above the error bars denote statistically significant differences between the treatments within each specific strain, as determined by Tukey’s HSD post-hoc test (*p* < 0.05).

The analysis of metal accumulation in Pdj and Po fungal strains revealed distinct, element-specific responses to the applied fortifications (Control denotes the culture medium without additional mineral supplementation, FA200 indicates supplementation with the respective element at a concentration of 200 mg/L, FA500 indicates supplementation at 500 mg/L, and Mix denotes the multielement treatment containing Ca, Mg, and Zn, as depicted in Figure 2).

For calcium, the highest accumulation in both strains was observed in the 500 mg treatment (Fa500), reaching estimated model means of 98.34 for *Pdj* and 98.94 for *Po*, which were significantly higher (group ‘c’) than all other treatments according to the Tukey HSD test. The 200 mg dose (FA200) and multi-element Mix also significantly increased calcium levels (group ‘b’) compared to the unfortified Control (group ‘a’). Conversely, magnesium accumulation displayed a very different pattern; the 500 mg fortification did not significantly increase Mg levels compared to the Control, with both falling into the lowest statistical group ‘a’ across both strains. Instead, the highest magnesium accumulation was achieved using the lower 200 mg dose and the Mix, which formed the significantly higher group ‘b’. Regarding zinc, both strains exhibited a clear dose-dependent increase, with the Fa500 and Mix treatments promoting the highest accumulation (group ‘c’), significantly outperforming the FA200 treatment (group ‘b’) and the unfortified Control (group ‘a’). These statistical differencesindicate that while a 500 mg dose or a multi-element mixture maximizes zinc and calcium uptake, magnesium accumulation is optimal at a lower 200 mg dose or in the mixture, suggesting that high individual doses of magnesium may actually inhibit its own accumulation.

The distinct, element-specific accumulation profiles observed in *P. djamor* and *P. ostreatus* reveal complex, highly regulated metal uptake mechanisms within the fungal mycelium [40,41,42]. The robust, dose-dependent accumulation of calcium and zinc—peaking at the highest applied dose (500 mg)—indicates that these *Pleurotus* strains possess a high tolerance for heavy and alkaline earth metals [43], likely sequestering them efficiently via cell wall biosorption (e.g., binding to chitin and β-glucans) or via vacuolar compartmentalization to prevent cellular toxicity [44,45,46,47]. Conversely, the biphasic response to magnesium, where the 500 mg dose failed to increase intracellular levels while the 200 mg dose succeeded, suggests a strict negative feedback mechanism [33]. In fungal physiology, exposure to excessive extracellular magnesium can trigger the rapid downregulation or internalization of specific divalent cation transporters to prevent osmotic stress and metabolic disruption [48]. Interestingly, the multielement mixture successfully promoted significant accumulation across all three elements, circumventing the magnesium inhibition seen in the high-dose singular treatment [41,49]. This phenomenon may result from competitive interactions at the transport sites or a more balanced electrochemical gradient across the plasma membrane, preventing the cellular shock associated with isolated high-magnesium exposure [50,51,52]. These findings demonstrate that while *Pleurotus* biomass is an excellent matrix for multielement biofortification, the formulation of the enriching media must be carefully balanced—utilizing multi-mineral mixtures rather than excessive singular doses—to optimize the nutritional and therapeutic profile of the resulting wound dressing material.

### 2.3. Textural and Morphological Properties of Biomass

**Figure 3:**
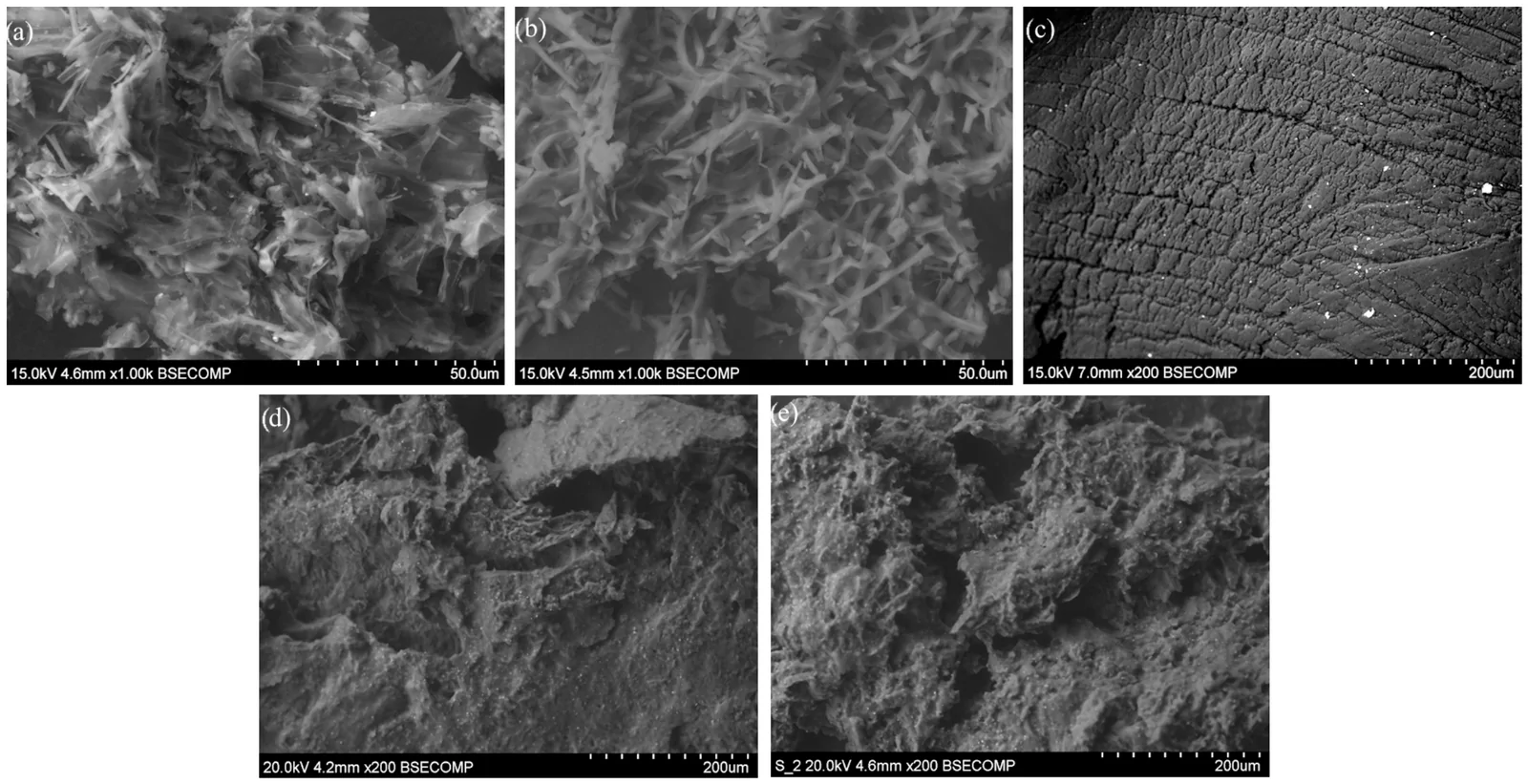
SEM analysis of biomass material: (**a**) Pdj—*P. djamor*, (**b**) Po—*P. ostreatus*, (**c**) γ-PGA—γ-PolyGlutamic Acid, (**d**) Pdj + PGA—*P. djamor* + γ-PolyGlutamic Acid, (**e**) Po + PGA—*P. ostreatus +* γ-PolyGlutamic Acid.

The textural properties of the control biomasses of *Pleurotus ostreatus* (Po) and *P. djamor* (Pdj), their PGA-enriched counterparts, and pure poly(γ-glutamic acid) (PGA) were evaluated using nitrogen adsorption and BET analysis, and the obtained results were interpreted together with SEM observations (Figure 3).

The control samples exhibited low BET-specific surface areas, equal to 1.3214 m^2^/g for Po and 1.0319 m^2^/g for Pdj, indicating poorly developed porous structures typical of non-carbonized biomass-based materials [53].

The SEM micrographs of the control biomasses confirmed clear differences in the surface organization between the two *Pleurotus* species. The Po sample displayed a relatively open and heterogeneous network of intertwined hyphal elements with visible voids and inter-fibrillar spaces, whereas the Pdj biomass appeared more compact, irregular, and locally agglomerated. These morphological differences are consistent with the BET results, since the more open Po structure corresponded to the highest specific surface area among the tested fungal biomasses, while the denser Pdj morphology was associated with a lower accessible surface.

The addition of PGA affected both the morphology and textural parameters of the biomasses in a species-dependent manner. In the Po-PGA sample, the fibrillar framework remained visible, although the structure became more compact and partially fused by a continuous matrix. Accordingly, only a slight decrease in BET surface area was observed, from 1.3214 to 1.1401 m^2^/g, suggesting that PGA did not substantially block the nitrogen-accessible surface of *P. ostreatus*. A markedly different effect was noted for the Pdj-PGA material. SEM images showed a much denser and more continuous surface, with strongly reduced visibility of individual hyphal elements and fewer open inter-structural voids. This observation was accompanied by a pronounced reduction in BET surface area to 0.2872 m^2^/g, indicating reduced accessibility of the material to nitrogen molecules after γ-PGA modification. Together with the denser morphology observed by SEM, this finding is consistent with a γ-PGA-associated structural rearrangement and/or partial masking of accessible inter-hyphal regions. However, BET analysis alone does not provide direct evidence of γ-PGA coverage of the hyphal surface or filling of specific inter-structural spaces [28]. Pure PGA exhibited a compact, aggregated, and non-fibrillar morphology with a folded and irregular surface containing local cracks and shallow depressions. This dense structure corresponded well to its low BET surface area of 0.3112 m^2^/g. Importantly, the close similarity between the morphology and BET values of pure PGA and the Pdj-PGA sample suggests that, in the *P. djamor* system, PGA dominated the surface characteristics of the resulting material to a much greater extent than in the *P. ostreatus* system [54].

Overall, the combined SEM and BET results demonstrated that PGA supplementation did not improve the textural development of the tested biomasses. Instead, especially for *P. djamor*, the additive promoted the formation of a denser and less accessible surface structure. Similar relationships between surface compaction observed by SEM and reduced accessible surface area determined by BET have been reported for other biomass-derived materials, where adsorption-related properties depend not only on the total surface area but also on pore accessibility, structural arrangement, and surface chemistry.

### 2.4. Surface Wettability Analysis

The wettability of fungal biomass was evaluated for unmodified samples and samples modified with γ-polyglutamic Acid (γ-PGA). Biomass derived from two *Pleurotus* species, *P. djamor* (Pdj) and *P. ostreatus* (Po), was investigated. Surface interactions were assessed using two probe liquids with different polarities: distilled water (H_2_O) and bovine serum.

**Figure 4:**
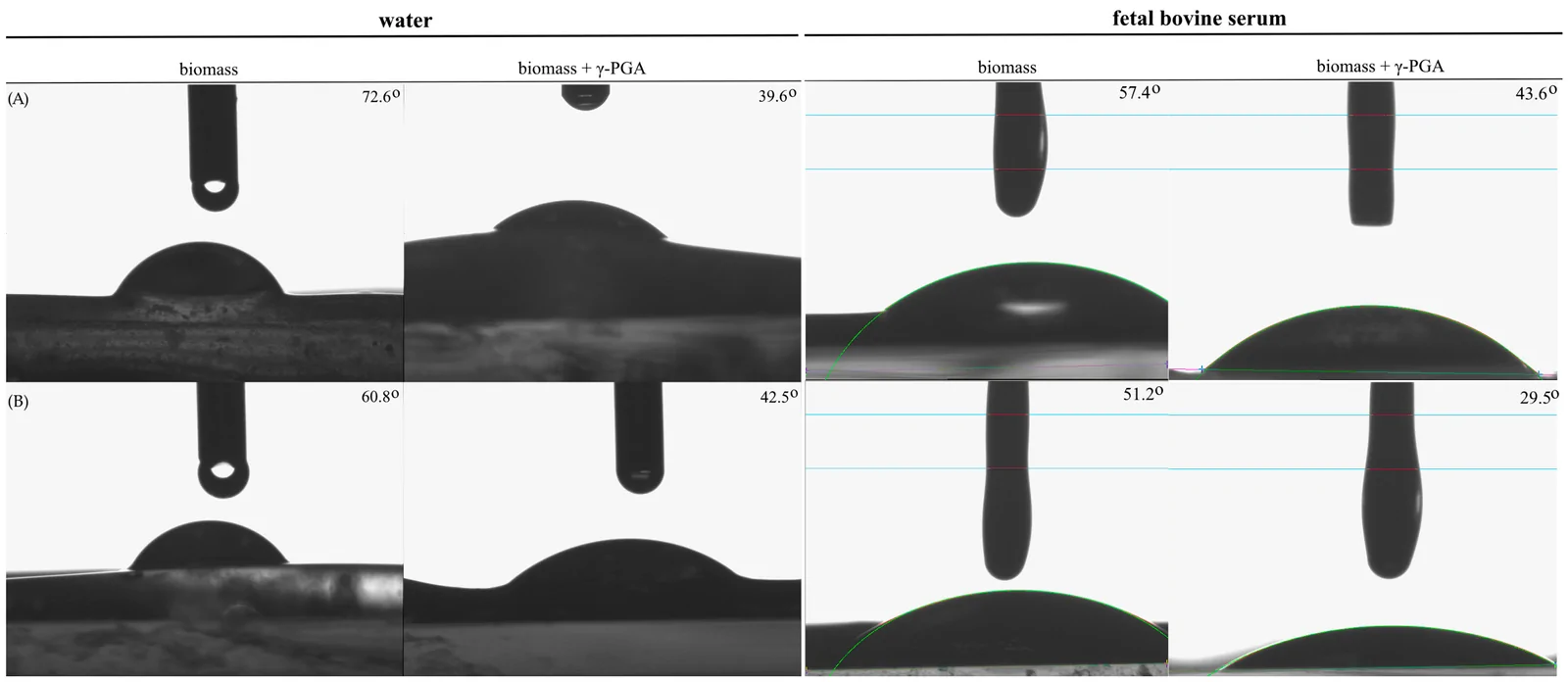
Static contact angle of water and bovine serum measurements on fungal biomass: (**A**) *P. djamor* control and (**B**) *P. ostreatus*.

For the unmodified biomass, the water contact angles were 60.8° for Po and 72.6° for Pdj. The incorporation of γ-PGA reduced the static contact angle in comparison with the corresponding untreated biomass, irrespective of the liquid applied. These results indicate that γ-PGA functionalization increased the wettability of fungal biomass surfaces (Figure 4). This result can be associated with the chemical structure of γ-PGA, which contains hydrophilic carboxyl and amide groups that can interact with polar liquids [31]. Their presence on the biomass surface may increase the polar component of the surface free energy, thereby promoting the spreading of the liquid drop [55]. The effect may also depend on species-specific differences in cell-wall composition, surface roughness, porosity, and the accessibility of functional groups in the fungal biomass [56].

The reduction in the contact angle following γ-PGA incorporation indicates that γ-PGA functionalization increased the hydrophilicity and wettability of the biomass surface. For water, the static contact angles of the γ-PGA-modified samples were 42.5° for Po and 39.6° for Pdj. The lower value recorded for Pdj may indicate more effective surface coverage with γ-PGA or a greater availability of polar groups at the material–liquid interface. Nevertheless, the final wettability of the modified samples likely reflects both the chemical contribution of γ-PGA and changes in surface morphology resulting from the modification process [57].

**Figure 5:**
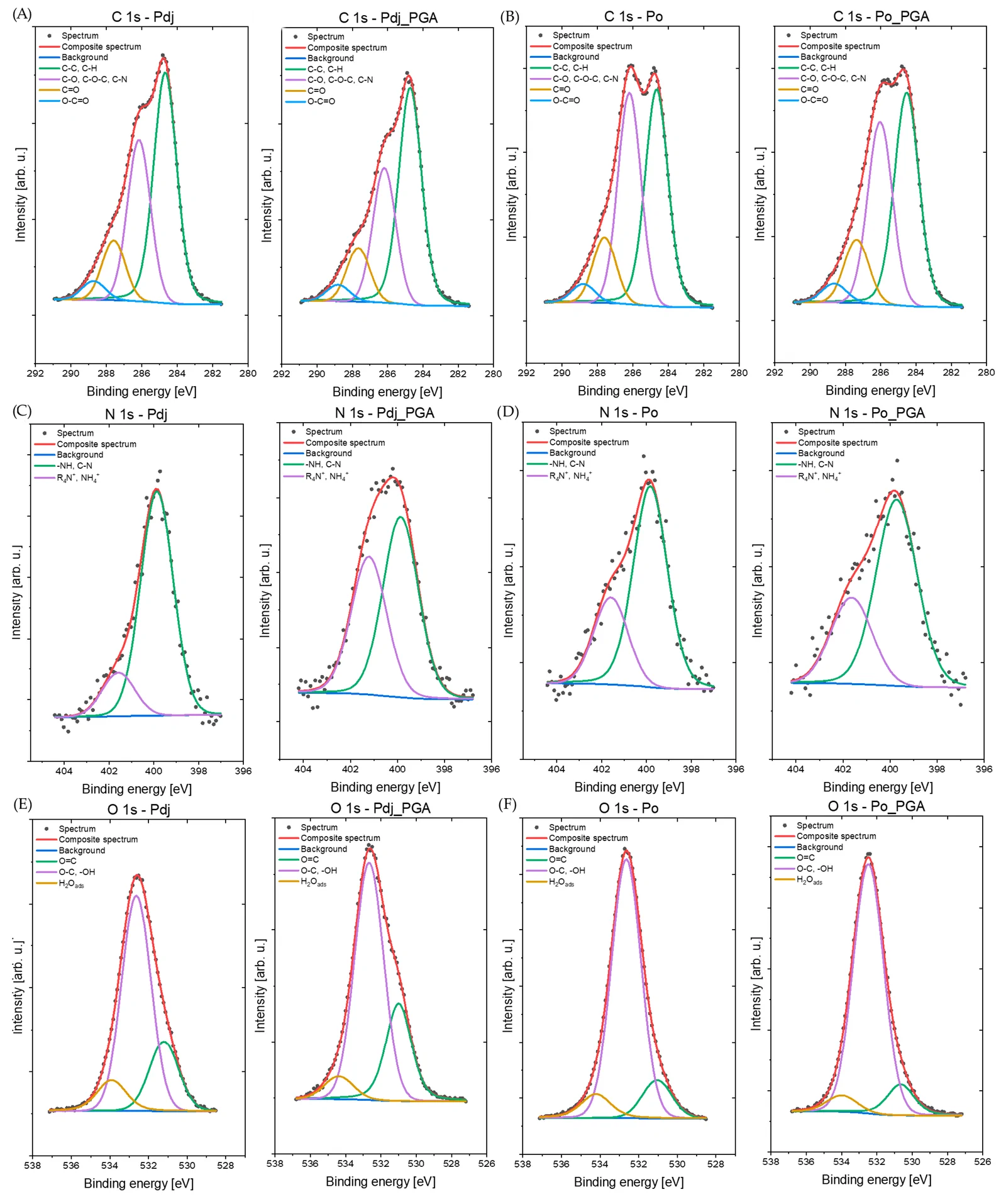
High-resolution XPS spectra of the surfaces of *Pleurotus djamor* (Pdj) and *Pleurotus ostreatus* (Po) biomass cultured in basal medium and medium supplemented with γ-polyglutamic acid (γ-PGA): (**A**,**B**) C 1s, (**C**,**D**) N 1s, and (**E**,**F**) O 1s regions. The fitted components corresponded to carbon- and oxygen-containing functionalities characteristic of fungal cell wall biopolymers and γ-PGA-associated functional groups.

A comparable pattern was observed when bovine serum was used as the testing liquid. The unmodified biomass exhibited static contact angles of 51.2° for Po and 57.4° for Pdj. After modification with γ-PGA, these values decreased to 29.5° and 43.6°, respectively (Figure 5). The lower values obtained with bovine serum than with water may be related to the complex composition of the serum, including proteins, lipids, and electrolytes, which can interact with the heterogeneous fungal surface [58]. The substantial decrease observed for Po after γ-PGA modification suggests an enhanced affinity of this biomass for aqueous, biologically relevant media. Such behavior may be advantageous in applications requiring rapid hydration, liquid absorption, or contact with biological fluids.

### 2.5. XPS Analysis

X-ray photoelectron spectroscopy (XPS) was used to examine the surface chemical composition and functional groups of the in vitro biomass of *Pleurotus ostreatus* (Po) and *Pleurotus djamor* (Pdj), cultivated in the basal medium and medium supplemented with γ-polyglutamic acid (γ-PGA). The survey spectra of all samples were dominated by carbon, oxygen, and nitrogen, confirming the organic and biopolymeric nature of the fungal biomass. The detected surface composition was consistent with the coexistence of polysaccharides, including glucans and chitin-related structures, as well as proteinaceous components. The surface chemical composition determined by XPS curve fitting is summarized in Table 1.

In non-supplemented cultures, carbon was the predominant element on the biomass surface. The total carbon contribution, calculated from the C 1s components, was 69.5 at.% for Pdj and 65.9 at.% for Po. The surfaces of both control biomasses were also characterized by substantial oxygen contents, reaching 26.5 at.% for Pdj and 30.9 at.% for Po, whereas nitrogen accounted for 4.3 and 2.4 at.%, respectively. Thus, *P. djamor* biomass exhibited a relatively higher nitrogen contribution, whereas *P. ostreatus* surface was comparatively more oxygenated.

The high-resolution C 1s spectra were deconvoluted into four components located at approximately 284.8, 286.2, 287.6, and 288.8 eV, attributed to C–C/C–H, C–O, C=O, and O–C=O groups, respectively. In both control samples, the major contributions of C–O and carbonyl/carboxyl-containing species indicate the presence of hydroxyl-rich polysaccharide structures and oxidized carbon functionalities typical of fungal cell wall constituents. Notably, Po contained a higher contribution of C–O groups than Pdj (26.2 vs. 22.8 at.%), whereas Pdj showed a greater proportion of C–C/C–H species (35.6 vs. 29.3 at.%). Representative high-resolution C 1s, N 1s, and O 1s spectra of the control and γ-PGA-supplemented fungal biomasses are presented in Figure 5.

Supplementation with γ-PGA modified the surface composition of both fungal biomasses. For Pdj-PGA, the total carbon and oxygen contents were 68.6 and 26.1 at%, respectively, while nitrogen increased to 4.8 at.%, respectively. In contrast, Po-PGA contained 66.8 at.% carbon, 29.7 at.% oxygen, and 2.6 at.% nitrogen. Compared with the respective controls, γ-PGA supplementation increased the nitrogen signal in both species from 4.3 to 4.8 at.% in *P. djamor* and from 2.4 to 2.6 at.% in *P. ostreatus*. This observation supports the incorporation, adsorption, or surface association of γ-PGA-derived nitrogen-containing functionalities with fungal biomass.

The O 1s components showed only moderate, species-dependent changes after γ-PGA functionalization. In Pdj, the contribution assigned to hydroxyl/ether groups remained essentially unchanged (17.3 at.% for Pdj and 17.2 at.% for Pdj-PGA), whereas the carbonyl oxygen contribution increased from 5.7 to 7.2 at.%. In Po, the carbonyl oxygen contribution decreased slightly from 3.7 to 3.2 at.%, while the hydroxyl/ether component remained nearly unchanged (24.5 and 24.8 at.% for Po and Po-PGA, respectively). These small changes should be interpreted cautiously, as they may reflect both the surface association of γ-PGA and the inherent chemical heterogeneity of fungal biomass. The N 1s spectra contained a principal component at approximately 399.9 eV, assigned to amine and amide groups, and a minor component at approximately 401.6 eV, attributed to protonated nitrogen species. The increase in the signal associated with amine/amide functionalities after γ-PGA enrichment was especially evident for Pdj-PGA, in which the N 1s contribution at 399.9 eV increased from 3.6 to 4.1 at.%. Since γ-PGA is a polyamide-type biopolymer composed of glutamic acid residues, this change is consistent with the contribution of amide-containing γ-PGA chains to the surface chemistry of the obtained fungal material.

Minor amounts of phosphorus, sulfur, and chloride were also detected. Phosphorus was present at 0.3–0.4 at.% and was assigned to phosphate-containing species, potentially associated with phospholipids or other cellular metabolites. Sulfur was detected at low levels, ranging from 0.1 to 0.2 at.%, and was attributed to oxidized sulfur species.

Overall, XPS demonstrated that γ-PGA supplementation caused measurable but moderate changes in the surface chemistry of both *P. ostreatus* and *P. djamor* biomasses. The enrichment-related increase in nitrogen-containing groups, particularly in *P. djamor*, together with changes in oxygenated carbon species, suggests an interaction between γ-PGA and fungal cell-wall components. Such modifications may be relevant for the subsequent use of γ-PGA-enriched fungal biomass as a functional biomaterial, as surface amide, carboxyl, hydroxyl, and ether groups can influence hydration, ion-binding capacity, interfacial interactions, and compatibility with polymeric matrices. While XPS, alongside SEM and BET analyses, effectively confirmed the surface presence and chemical association of γ-PGA with the fungal biomass, these techniques are primarily surface-sensitive. They do not quantify the internal spatial distribution of the polymer throughout the bulk mycelial matrix nor its retention stability following mechanical washing. The gravimetric assessment indicated an initial estimated polymer loading efficiency of approximately 10%; however, further quantitative bulk mapping and retention assays will be essential to fully characterize the structural uniformity of the modified material.

### 2.6. Thermal Properties of Fungal Biomass

#### 2.6.1. DSC Analysis

##### Unmodified Biomass

**Figure 6:**
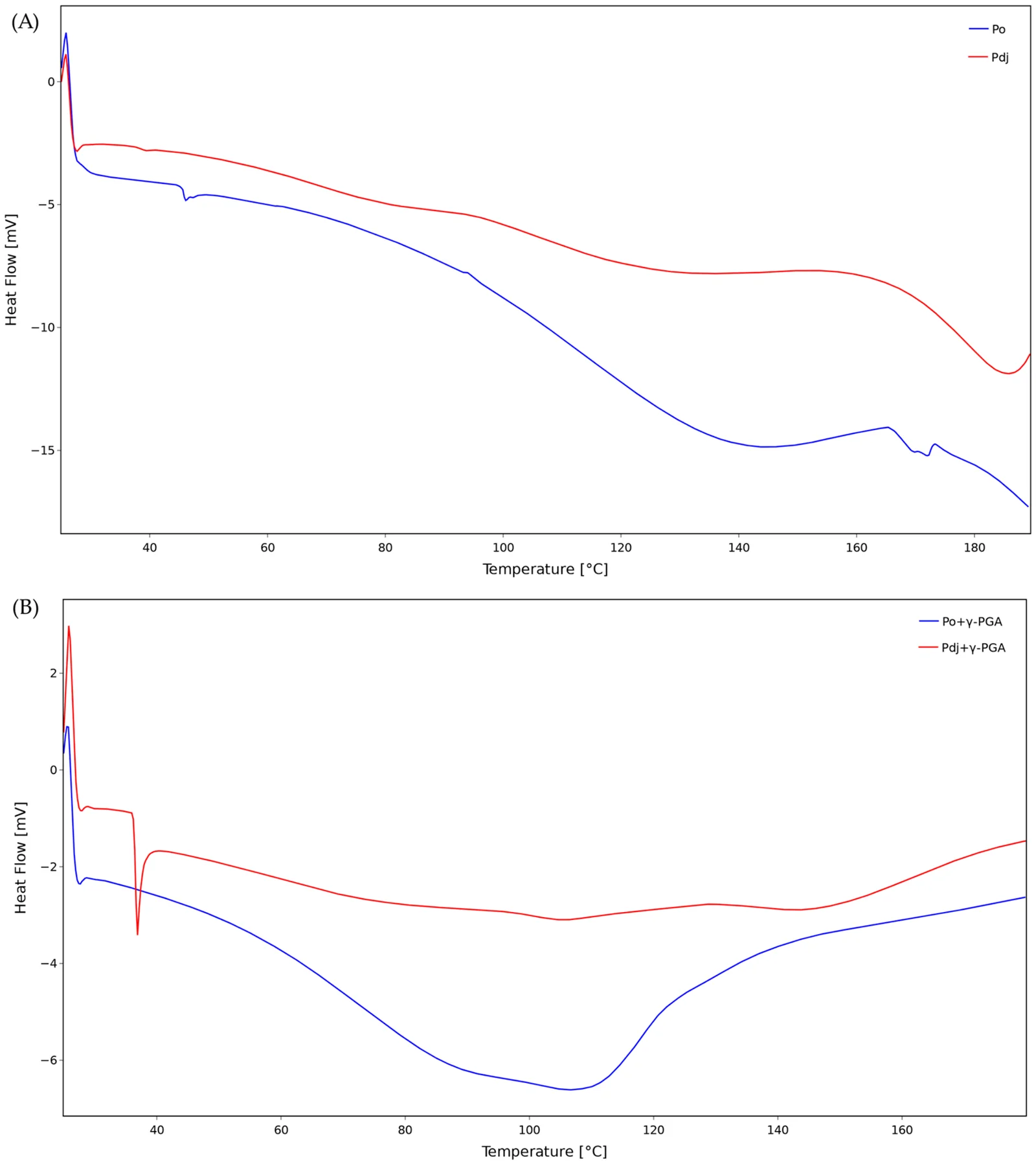
DSC thermograms of unmodified (**A**) and γ-PGA-modified (**B**) fungal biomass: *Pleurotus ostreatus* (Po), *Pleurotus djamor* (Pdj), Po + γ-PGA, and Pdj + γ-PGA. The heat flow was recorded as a function of temperature.

The thermal behavior of unmodified and γ-PGA-modified fungal biomasses was evaluated using DSC analysis. The DSC thermograms are presented in Figure 6.

The DSC profiles of the unmodified biomasses of *Pleurotus ostreatus* (Po) and *Pleurotus djamor* (Pdj) revealed thermally responsive multicomponent lignocellulosic–fungal matrices. Both thermograms showed a low-temperature thermal event beginning near the lower measurement limit (approximately 25 °C), which can be associated primarily with the release of physically adsorbed and weakly bound water. Such behavior is expected for fungal biomass because hydrophilic polysaccharides, including chitin/chitosan, β-glucans, and other cell wall constituents, provide numerous sites for water association [59,60].

The Po sample exhibited a higher initial heat-flow response than Pdj, reaching approximately 2.0 mW near 25.8 °C, whereas the corresponding maximum for Pdj was lower, at approximately 1.1 mW around 25.8 °C. This difference suggests that Po contained a larger fraction of readily mobilized water and/or a distinct organization of hydrophilic cell-wall components. Since the dataset represents a single DSC run and no replicate-derived thermodynamic parameters were supplied, this observation should be regarded as comparative rather than statistically confirmed.

The different profiles may also reflect species-specific variations in the composition and supramolecular arrangement of fungal polysaccharides, proteins, lipids, and residual substrate-derived components. The relatively more pronounced thermal response of Po may be attributed to the matrix undergoing greater molecular relaxation or dehydration in the low-temperature region, while the weaker signal for Pdj may indicate lower free-water content or stronger water–biopolymer interactions.

##### Modified Biomass

The incorporation of γ-PGA changed the DSC responses of both fungal biomasses, demonstrating that the modifier affected the thermal behavior of the fungal matrices. In Po + γ-PGA, the initial heat-flow maximum was approximately 0.9 mW at approximately 25.5–25.8 °C, which was markedly lower than that observed for unmodified Po. The decrease in the low-temperature signal suggests that γ-PGA reduced the contribution of freely removable moisture and promoted stronger interactions between water, anionic poly(amino acid), and biomass constituents.

This result can be interpreted as evidence of improved molecular integration between γ-PGA and the *P. ostreatus* matrix during the fermentation process. The carboxyl groups of γ-PGA can participate in hydrogen bonding and ionic interactions with hydroxyl- and amino-containing groups present in fungal cell wall polymers, potentially restricting chain mobility and modifying water distribution within the material [31,61]. Consequently, Po + γ-PGA formed a more thermally coherent matrix than the corresponding unmodified biomass.

In contrast, Pdj + γ-PGA showed a considerably stronger initial heat-flow signal than unmodified Pdj, with a maximum of approximately 3.0 mW near 25.8 °C. This pronounced response indicates that γ-PGA increased the fraction of thermally active bound or weakly associated water in the *P. djamor* system or induced a more extensive rearrangement of the polymer–water network during the early heating stage. The divergent response of Po and Pdj after γ-PGA addition emphasizes that the compatibility between γ-PGA and fungal biomass depends strongly on the species-specific biochemical composition and microstructural organization of the substrate.

Overall, γ-PGA did not produce a uniform thermal effect across the two biomasses. In Po, the modification attenuated the low-temperature thermal event, indicating reduced molecular mobility and/or lower availability of labile water. Conversely, in Pdj, γ-PGA intensified this event, suggesting enhanced hydration or a less compact polymeric network. These findings indicate that γ-PGA can be used to tailor the thermal behavior of fungal biomass-based materials; however, the final effect depends on the intrinsic properties of the fungal species.

#### 2.6.2. TG Analysis

##### Unmodified Biomass

**Figure 7:**
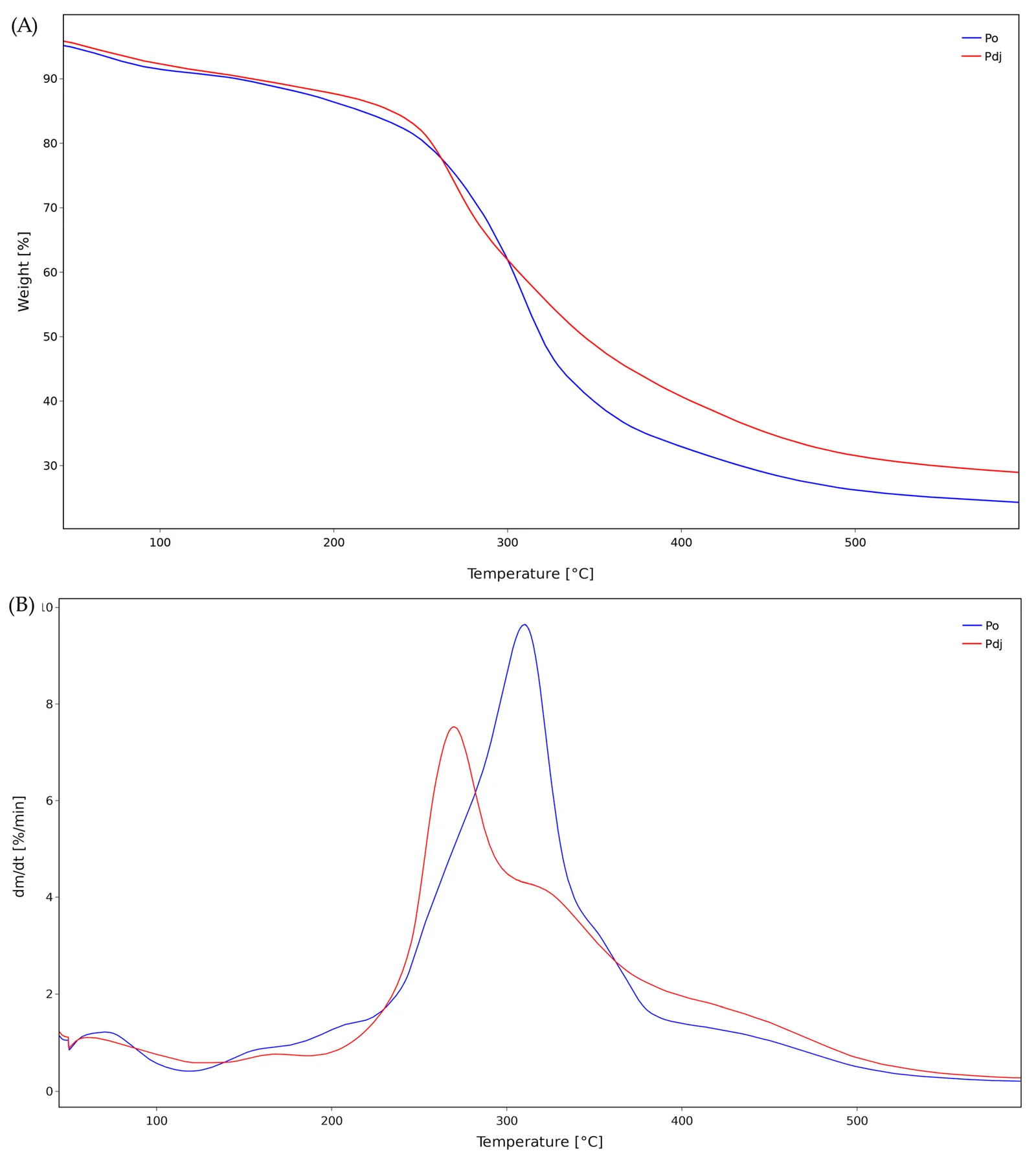
Comparison of the TG (**A**) and DTG (**B**) curves obtained for the unmodified biomass *P. djamor* (Pdj) and *P. ostreatus* (Po), showing the relationship between weight loss and temperature.

The TG profiles of the unmodified Po and Pdj samples revealed a multistep degradation pattern, indicating the presence of several thermally distinct fractions within the biomass. In both materials, the initial mass loss observed at lower temperatures can be attributed to the removal of physically adsorbed water and weakly bound moisture, which is typical for hydrophilic biomaterials containing polar functional groups (Figure 7).

At higher temperatures, both Po and Pdj underwent the principal degradation stage associated with the decomposition of the organic matrix [62,63,64]. This region likely reflects the breakdown of low-molecular-weight constituents and less stable structural components, followed by the progressive degradation of more condensed organic matter. The DTG traces suggest that these transformations were not fully resolved in a single step, indicating the chemical heterogeneity of the biomass and overlapping decomposition events.

A further decrease in mass in the upper temperature range may be attributed to the decomposition of more thermally stable fractions and the slow degradation of carbonaceous residues formed during earlier stages of heating. The final residue indicates that both unmodified samples retained a non-volatile fraction after thermal treatment, which may reflect the presence of mineral constituents and char-forming components. Differences between Po and Pdj in the shape of the TG/DTG curves suggest that, despite their broadly similar degradation pathway, the relative contribution of individual components and their thermal resistance were not identical.

##### Modified Biomass

**Figure 8:**
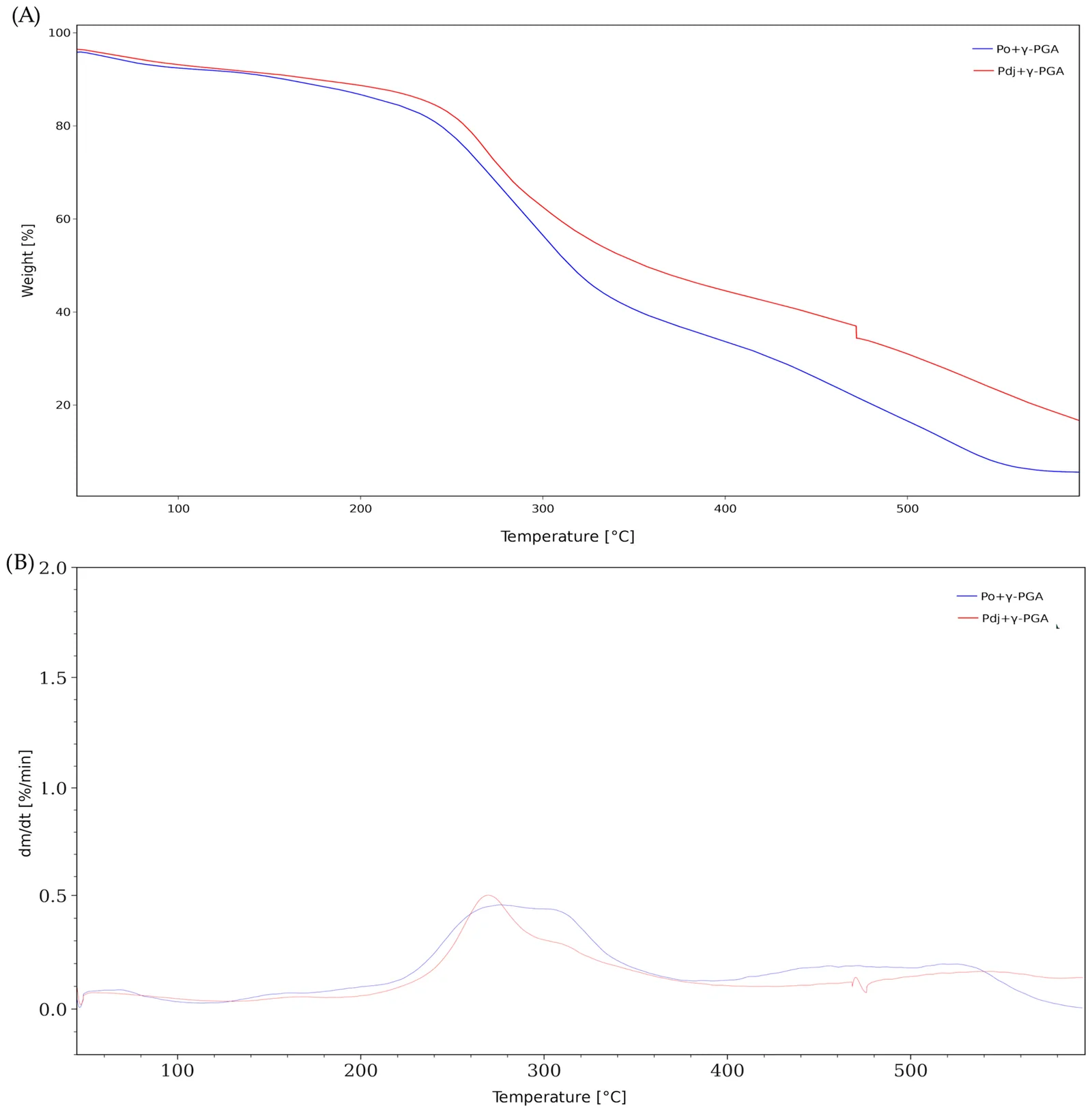
Comparison of the TG (**A**) and DTG (**B**) curves obtained for the modified biomass *P. djamor* + γ-PGA (Pdj + γ-PGA) and *P. ostreatus* + γ-PGA (Po + γ-PGA), showing the relationship between weight loss and temperature.

The introduction of γ-PGA altered the thermal behavior of both biomass samples, as evidenced by changes in the course of the TG and DTG curves for Po + γ-PGA and Pdj + γ-PGA. The modified samples exhibited a multistage decomposition pattern; however, the intensity and distribution of the mass-loss events differed from those observed for the unmodified materials. This behavior indicates that the modifier did not simply act as an inert additive but participated in the thermal transformation of the system (Figure 8).

The more pronounced effects in the low- and intermediate-temperature regions may be related to the hydrophilic and polyelectrolytic nature of γ-PGA, which can enhance water binding and introduce additional thermally labile groups into the material. Because γ-PGA contains numerous carboxyl functionalities, its presence may promote overlapping degradation steps associated with dehydration, decarboxylation, and polymer chain decomposition [65,66]. Consequently, the modified samples may exhibit broader DTG maxima or shifts in the temperatures of the principal degradation steps relative to the native biomass.

The effect of γ-PGA was not identical for Po and Pdj, which suggests that the modifier interacted differently with each biomass matrix. These differences may result from variations in the original composition of the samples and distinct intermolecular interactions, such as hydrogen bonding or electrostatic interactions, between γ-PGA and matrix components. Overall, the TG results indicate that γ-PGA incorporation changed the thermal decomposition pathway of both biomasses and affected their thermal stability profile, particularly in the dehydration region and during the main decomposition step.

A comparison of the TG/DTG profiles showed that the addition of γ-PGA modified the thermal-degradation behavior of both Po and Pdj biomass samples. The modified materials displayed altered mass-loss profiles, particularly in the low- and intermediate-temperature regions, which may be related to the hydrophilic character and carboxyl-rich structure of γ-PGA. The changes in the position and/or broadening of the DTG signals suggest overlapping thermal transformations of the biomass components and the polymeric modifier. Moreover, the effect of γ-PGA was not identical for Po and Pdj, indicating differences in the interactions between the modifier and the respective biomass matrices. Overall, γ-PGA incorporation changed the degradation pathway of the materials and affected their thermal stability.

### 2.7. Cytotoxicity of the Material

To isolate the specific biological influence of the polymeric modifier, the cytotoxicity and antimicrobial assays in this study were conducted using the unfortified base fungal matrices. While our prior analyses demonstrated that the biomass can be successfully enriched with zinc, calcium, and magnesium, evaluating the independent cytoprotective and antimicrobial contributions of these specific accumulated metals requires dedicated comparative studies and release-kinetic assays, which remain a subject for future investigation.

**Figure 9:**
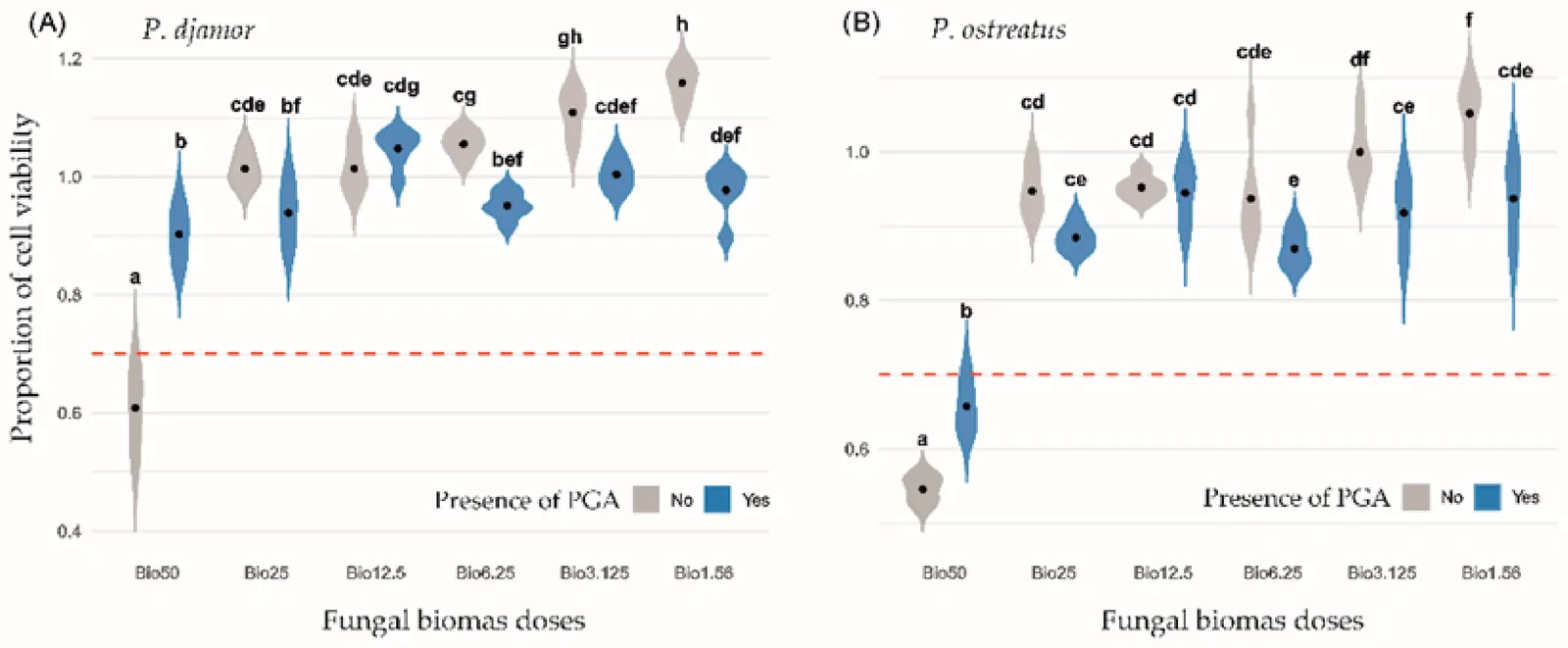
Violin plots presenting the viability of human HaCaT keratinocytes exposed to *Pleurotus djamor* (**A**), Pdj) and *Pleurotus ostreatus* (**B**), Po) base extracts at varying doses (Bio50–Bio1.56). The plots compare the unfortified fungal matrices (No, grey) with those fortified with γ-olylutamic acid (γ-PGA) (Yes, blue). Black dots within the violins represent the model-estimated mean viability. The horizontal red dashed line denotes the 70% cell viability threshold, according to the ISO 10993-5:2009 standard. Different lowercase letters above the distributions indicate statistically significant differences (*p* < 0.05) across all treatments within a given strain, as determined by a generalized linear mixed model (GLMM) followed by Tukey’s HSD post-hoc test.

Generalized linear modeling revealed a significant three-way interaction between fungal strain, biomass dose, and addition of γ-PGA on cell viability (F = 8.44, *p* < 0.001), driven primarily by a highly significant dose-dependent response to the functional polymer (Dose × PGA interaction: F = 65.61, *p* < 0.001) (Figure 9). High amounts of unfortified fungal biomass, specifically at the Bio50 dose, exhibited notable cytotoxicity, causing cell viability to drop to approximately 60% (95% CI) for Pdj and 52% (95% CI) for Po, decisively falling below the 70% biological safety threshold outlined by the ISO 10993-5:2009 standard [67]. However, the application of γ-PGA provided a biologically critical rescue effect at this highest biomass concentration, elevating cell viability by an absolute margin of 30–35%, pushing it back to approximately 90% and effectively shifting the material from a cytotoxic to a cytocompatible profile. Interestingly, at lower biomass concentrations (Bio3.125 and Bio1.56), the addition of γ-PGA statistically suppressed natural cell proliferation compared to the unfortified base matrices (*p* < 0.001). However, the biological significance of this suppression is minimal, as absolute cell viability in these groups remained well above 90%, posing no threat to cytocompatibility.

The complex, dose-dependent interplay between the *Pleurotus* matrix and γ-PGA reveals a crucial cytoprotective mechanism essential for the development of biocompatible wound dressings. The pronounced cytotoxicity observed at high doses (Bio50) of the unfortified fungal extracts likely stems from an overwhelming concentration of fungal secondary metabolites, such as terpenoids, specific proteins, or phenolic compounds, which can induce acute osmotic or oxidative stress in human keratinocytes. However, the incorporation of γ-PGA successfully neutralized this toxicity, rescuing cell viability above the ISO 10993-5:2009 70% safety threshold. This remarkable buffering capacity aligns perfectly with recent studies highlighting the shielding properties of this biopolymer. For instance, Zając et al. (2022) demonstrated that γ-PGA functions as a vital protective barrier, preventing microbial extinction and supporting cellular survival under highly toxic, hydrocarbon-contaminated environmental conditions [68]. Similarly, Krakowska et al. (2025) reported that γ-PGA supplementation provides a robust protective effect for fungal biomass itself, sustaining cellular growth and viability even under severe heavy metal stress [69].

Interestingly, at lower extract doses where the raw matrix naturally supported unhindered cell proliferation, the addition of γ-PGA slightly dampened metabolic activity. This dual behavior—rescuing cells from acute toxicity at high doses while mildly suppressing proliferation at low doses—suggests that γ-PGA acts as a broad homeostatic stabilizer. This neutralizing phenomenon is directly corroborated by Galanty et al. (2025) [70], who found that fortifying botanical extracts with γ-PGA resulted in a weaker overall cytotoxic profile against various cellular models, indicating a general dampening effect on the acute bioactivity of the matrix [70]. In the context of wound healing, this stabilizing trade-off is highly advantageous; the γ-PGA polymer effectively creates a safe, controlled microenvironment that shields the delicate, regenerating wound bed from localized tissue damage while ensuring the safe delivery of fungal bioactive agents.

### 2.8. Antimicrobial Activity

**Figure 10:**
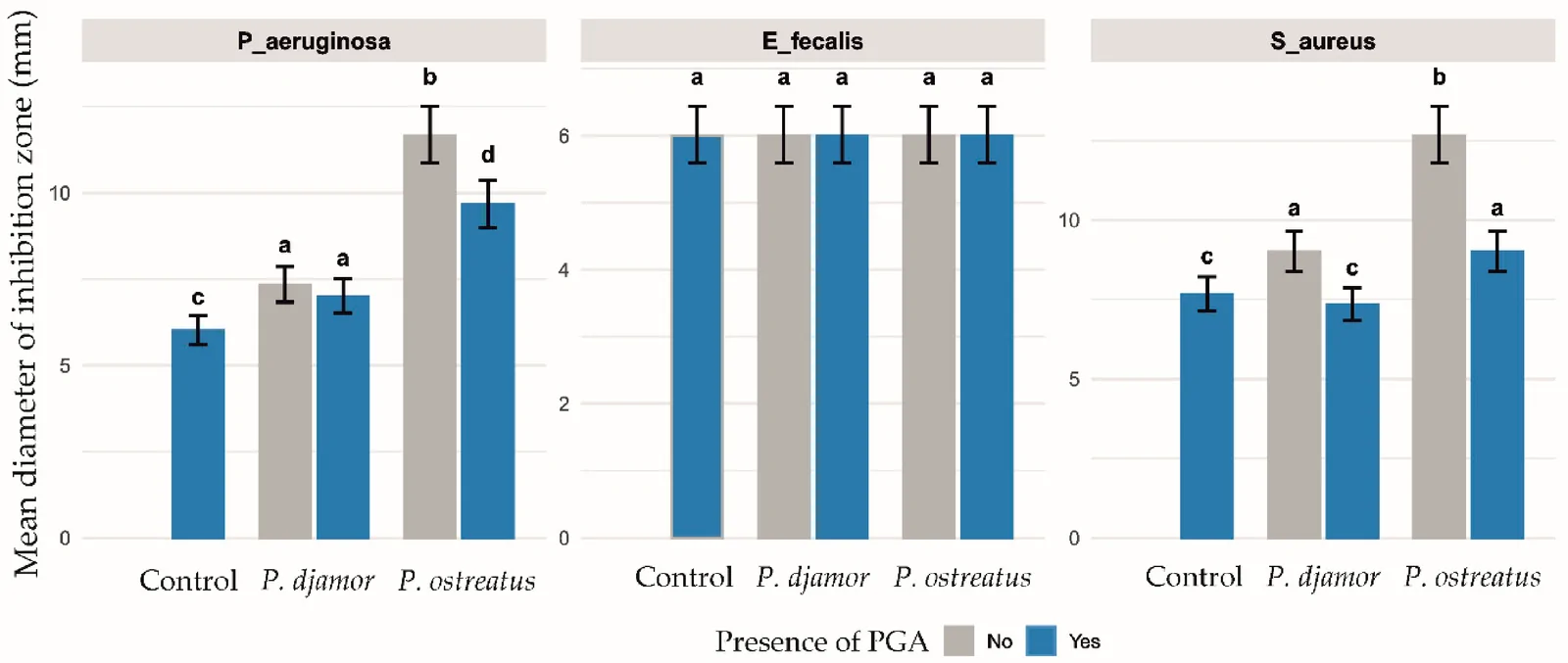
Antimicrobial activity expressed as mean inhibition zone diameters (mm) against three pathogenic bacterial strains: *P. aeruginosa*, *E. faecalis*, and *S. aureus*. Bar heights represent the marginal means estimated by a generalized linear mixed model (GLMM) utilizing a gamma distribution. The tested biomaterials include a control (Control) and matrices derived from *Pleurotus djamor* (Pdj) and *Pleurotus ostreatus* (Po) fungal biomass. The colors differentiate matrices prepared without (No, grey bars) and with (Yes, blue bars) the addition of γ-PGA. Vertical error bars indicate the 95% confidence intervals (CI) of the model. Different lowercase letters indicate statistically significant differences (*p* < 0.05) between the treatments within each specific bacterial strain, as determined by Tukey’s HSD post-hoc test.

The generalized linear mixed model (GLMM) analysis revealed that the diameter of the bacterial inhibition zones was significantly dependent on the bacterial target strain, applied fungal biomass, presence of gamma-PGA, and their two-way interactions (Target × Fungus: *p* < 0.001; Target × PGA: *p* < 0.001) (Figure 10). As visually confirmed in the panel for *E. fecalis*, this specific strain exhibited complete resistance to all tested variants, with the inhibition zone remaining consistently at the baseline 6.0 mm (statistical group ‘a’) regardless of the fungal strain or PGA addition. In contrast, the unfortified *Pleurotus ostreatus* (Po) biomass demonstrated the highest baseline antimicrobial activity against both *P. aeruginosa* (mean diameter of 11.66 mm, CI: 95%) and *S. aureus* (12.65 mm, CI: 95%). The addition of the γ-PGA polymer significantly reduced these inhibition zones to 9.66 mm and 9.00 mm, respectively. Biologically, this represents a moderate absolute reduction in macroscopic bacteriostatic efficacy of approximately 17% for *P. aeruginosa* and 29% for *S. aureus.* A similar suppressive effect of γ-PGA was observed for the Pdj strain against *S. aureus*, reducing the inhibition zone from 8.99 mm to 7.33 mm. While this attenuation is highly statistically significant (*p* < 0.001), its practical relevance must be contextualized within the T.I.M.E. wound-healing paradigm. The polymer-modified matrix sacrifices a fraction of its acute, diffusable antimicrobial potency in exchange for a massive, clinically necessary gain in host–cell cytoprotection, maintaining sufficient bacteriostatic pressure while ensuring host tissue safety.

The antimicrobial tests revealed a complex dynamic between the innate bioactivity of fungal matrices and the structural properties of the modifying polymer [71]. Consistent with the existing literature on the therapeutic properties of mushrooms, the unfortified *P. ostreatus* biomass exhibited potent, broad-spectrum bactericidal activity against key opportunistic wound pathogens, notably *S. aureus* and *P. aeruginosa* [72,73,74]. However, the incorporation of γ-PGA resulted in a measurable attenuation of these inhibition zones, alongside an observed resistance from *E. faecalis* [17]. This dampening effect is likely driven by the physicochemical nature of γ-PGA. As a highly hydrophilic, polyanionic macromolecule, γ-PGA forms a dense, water-retaining matrix that can physically encapsulate or electrostatically bind the active fungal secondary metabolites (such as phenolics, terpenoids, and specific proteins) [75]. While this hydrogel-like barrier slows the rapid, acute diffusion of these antimicrobial agents into the surrounding environment—resulting in smaller inhibition zones in vitro—it is precisely this controlled-release mechanism that is responsible for rescuing human keratinocytes from acute cytotoxicity. However, this barrier effect also highlights a critical limitation of the disk diffusion method for evaluating polymer-bound systems, as the reduced zone diameters may reflect restricted diffusion rather than an absolute loss of antibacterial efficacy. Furthermore, standard quantitative methods such as the Minimum Inhibitory Concentration (MIC) broth microdilution assay are equally problematic for this material. Because the γ-PGA-modified biomass is an insoluble, hydrogel-forming matrix, it does not dissolve homogeneously in liquid media and creates significant turbidity, rendering standard optical density readings inaccurate. Therefore, both agar diffusion and broth MIC data must be interpreted with caution for such solid biomaterials, and future quantitative efficacy must instead be evaluated using solid-surface contact-killing assays or dynamic time–kill kinetics of the leachate.

Recent studies by Nguyen and co-workers provide an informative, although not directly equivalent, example of multifunctional metal-containing antimicrobial materials. In those studies, oregano essential oil (OEO)-loaded, metal-doped ZIF-8 nanoparticles coated with hyaluronic acid (HA) were developed primarily for microbial control on food-contact surfaces and fresh produce. The optimized OEO–Ag–ZIF-8–HA formulation achieved more than 5-log bacterial reductions on latex surfaces and a 3.39-log CFU/g reduction on baby arugula leaves, while a related OEO–Ag–ZIF-8–HA system showed MIC and MBC values of 125 and 375 μg/mL, respectively, against *Listeria monocytogenes* [76,77]. These results reflect the high antimicrobial potency of an engineered nanoplatform combining metal-ion-associated activity, the ZIF-8 framework, and the membrane-active constituents of oregano essential oil.

The material investigated in the present study differs fundamentally in both composition and design rationale. Rather than using an inorganic metal–organic framework loaded with an essential oil, we developed a fungal biomass-based matrix enriched with Ca, Mg, and Zn during cultivation and subsequently functionalized with γ-PGA. The intended role of γ-PGA was primarily to modulate the surface properties and cytocompatibility of the fungal biomass rather than to act as a potent antimicrobial agent. Accordingly, the addition of γ-PGA moderately reduced the inhibition-zone diameters observed in the disk-diffusion assay, while substantially improving the viability of HaCaT keratinocytes exposed to concentrated biomass extracts. This trade-off is relevant to wound-care material design, where excessive antimicrobial activity accompanied by host–cell toxicity may be undesirable.

Direct comparison of antimicrobial performance with the ZIF-8-based systems should nevertheless be made cautiously. The studies by Nguyen et al. evaluated engineered nanoparticle suspensions against L. monocytogenes using MIC/MBC determination and quantitative reductions on latex or leafy-vegetable surfaces, whereas the present work evaluated solid biomass-derived extracts by disk diffusion against *E. faecalis*, *S. aureus*, and *P. aeruginosa*. In addition, inhibition-zone diameter is strongly influenced by diffusion through agar and may underestimate the activity of polymer-associated or poorly soluble materials. Therefore, the present results do not demonstrate superior antimicrobial potency relative to OEO–metal-doped ZIF-8–HA nanomaterials. Instead, they establish that γ-PGA functionalization can improve the cytocompatibility of mineral-enriched *Pleurotus* biomass while preserving measurable in vitro antibacterial activity. This provides a distinct rationale for further development of the material as a bioactive ingredient for hydrated wound-dressing systems, where quantitative viable-count, time–kill, antibiofilm, release, and wound-relevant performance studies will be required.

In the context of chronic wound management and the T.I.M.E. paradigm [78,79,80], absolute pathogen eradication at the expense of host tissue viability is clinically counterproductive [81,82]. Therefore, the γ-PGA-modified matrix functions not as a harsh sterilizing agent, but as a sophisticated homeostatic platform [83,84]. The γ-PGA-modified material should currently be regarded as a promising bioactive biomass component with improved in vitro cytocompatibility and measurable bacteriostatic activity, rather than as a fully validated wound-dressing platform. A limitation of the present study is that the γ-PGA-functionalized, Ca/Mg/Zn-enriched fungal biomass was evaluated as a bioactive material ingredient rather than as a finalized wound-dressing system. Consequently, swelling capacity, water-retention behavior, moisture-vapor transmission rate, and mechanical stability under hydrated conditions were not assessed. These clinically relevant properties depend strongly on the composition and architecture of the final dressing matrix, including its polymer composition, cross-linking density, thickness, and porosity. In our ongoing work, the developed fungal biomass is being incorporated into hydrogel-based formulations, in which these wound-dressing performance parameters will be systematically evaluated.

## 3. Materials and Methods

### 3.1. Description of Research

#### The Scheme of the Experiment

**Figure 11:**
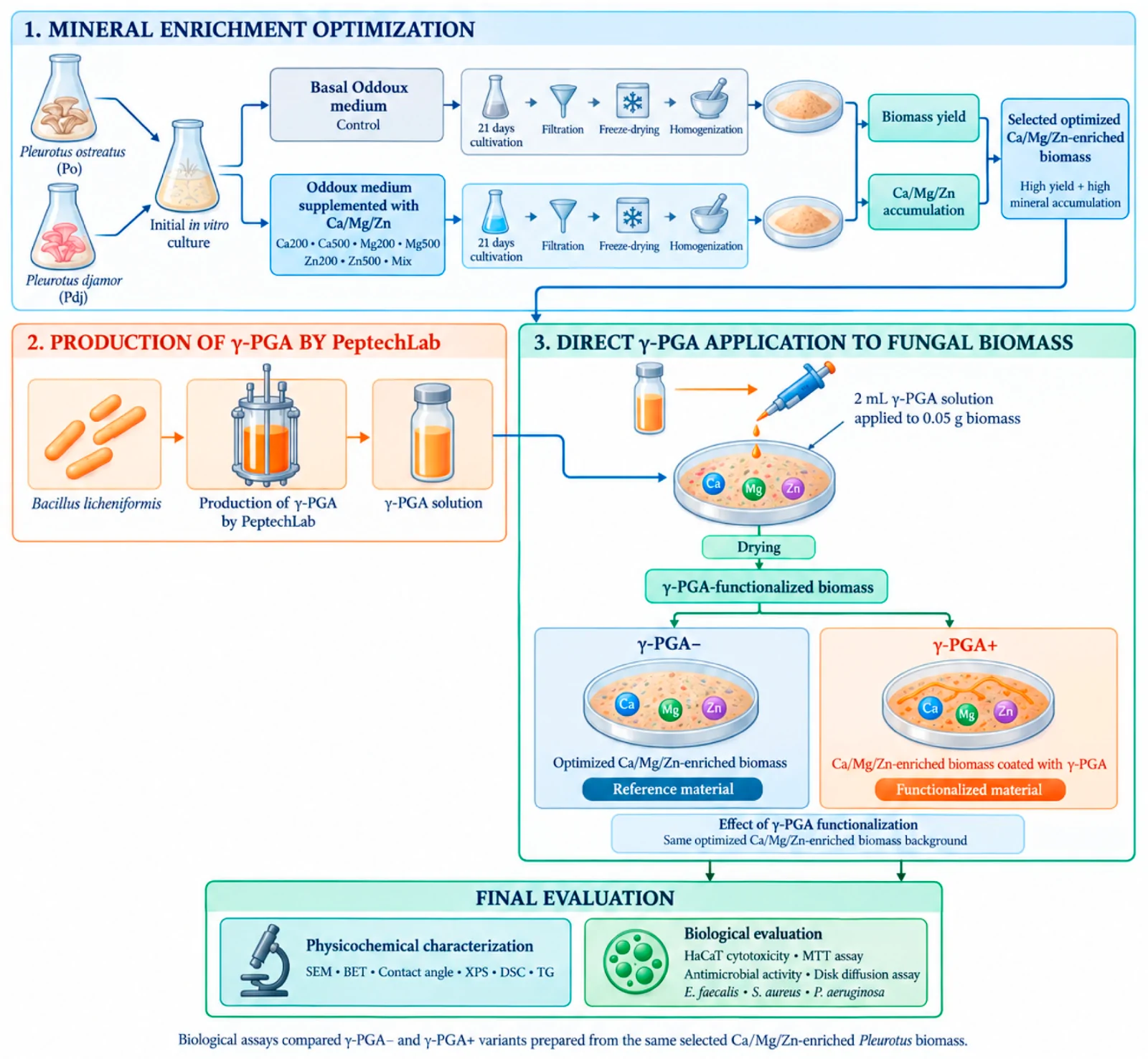
Schematic overview illustrating the principal stages and general design of this study.

To assess the potential of in vitro cultivated fungal biomass as a matrix for γ-PGA, this study was structured into four stages, each of which was further divided into specific sub-steps. The research scheme is shown in Figure 11.

- **STAGE 1** (Biomass production)

In vitro cultures of *P. djamor* and *P. ostreatus* were maintained in liquid Oddoux medium, either under controlled conditions or supplemented with Ca, Mg and Zn; after 21 days, the resulting biomass was subjected to filtration, freeze-drying, and homogenization to obtain dry mushroom biomass.

- **STAGE 2** (γ-PGA biosynthesis)

Purified γ-polyglutamic acid (γ-PGA), biosynthesized from *Bacillus licheniformis*, was acquired commercially (Peptechlab, Kraków, Poland) and prepared as an aqueous solution for subsequent biomass coating.

- **STAGE 3** (Biomass functionalization)

Dosing 2 mL of γ-PGA solution onto the surface of 0.05 g of freeze-dried mushroom biomass; the material was subsequently dried, yielding the final product in the form of biomass enriched with bioelements and coated with a γ-PGA layer.

### 3.2. Material Preparation

#### 3.2.1. *Pleurotus* spp. In Vitro Cultures Cultivation

Cultures of *Pleurotus ostreatus* (Po) and *Pleurotus djamor* (Pdj) were maintained in liquid Oddoux medium under controlled in vitro conditions. For each species and experimental treatment, 0.2 g of inoculum obtained from the respective starter culture was aseptically transferred into a 500 mL Erlenmeyer flask containing 250 mL of sterile liquid Oddoux medium [85].

The experimental design comprised an unfortified control and seven mineral-supplementation treatments: Ca200, Ca500, Mg200, Mg500, Zn200, Zn500, and Mix. The control medium received no additional calcium, magnesium, or zinc supplementation beyond the basal composition of the Oddoux medium. Calcium, magnesium, and zinc were supplied as calcium chloride (CaCl_2_), magnesium sulfate heptahydrate (MgSO_4_·7H_2_O), and zinc sulfate heptahydrate (ZnSO_4_·7H_2_O), respectively.

The numerical treatment codes 200 and 500 refer to the target concentrations of elemental Ca, Mg, or Zn in the culture medium, expressed in mg/L. Thus, Ca200 and Ca500 contained 200 and 500 mg/L elemental Ca, respectively; Mg200 and Mg500 contained 200 and 500 mg/L elemental Mg, respectively; and Zn200 and Zn500 contained 200 and 500 mg/L elemental Zn, respectively. The appropriate masses of CaCl_2_, MgSO_4_·7H_2_O, and ZnSO_4_·7H_2_O were calculated from the molar masses and elemental mass fractions of the respective salts before their addition to the culture medium.

The multielement treatment (Mix) contained elemental Ca, Mg, and Zn at final target concentrations of 200, 200, and 500 mg/L, respectively. These elements were supplied as CaCl_2_, MgSO_4_·7H_2_O, and ZnSO_4_·7H_2_O, respectively. The corresponding salt quantities were calculated to achieve these target elemental concentrations in the final culture medium.

Each species × treatment combination was prepared in five independent biological replicates, with each separately inoculated flask considered one experimental unit. Cultures were maintained for 21 days at 22 ± 2 °C under an illumination intensity of 200 lux and a light/dark regime of [insert the exact photoperiod applied]. Following cultivation, the biomass was separated from the liquid medium by filtration using a Pyrex Büchner funnel with a perforated plate (Merck, Darmstadt, Germany), freeze-dried at −40 °C using a FreeZone 4.5 freeze dryer (Labconco, Kansas City, MO, USA), and stored in a desiccator until further analyses.

##### Selection of the Mineral-Enriched Biomass for Biological Assays

Based on the preliminary optimization experiment, one mineral-enriched biomass variant was selected for subsequent γ-PGA functionalization and biological evaluation. The selection criterion was the combined achievement of high biomass yield and high accumulation of the investigated mineral elements. The selected biomass was obtained from cultures supplemented with Ca, Mg, and Zn at concentrations of 200 mg/L Ca, 200 mg/L Mg, and 500 mg/L Zn, respectively. This material is hereafter referred to as the Ca/Mg/Zn-enriched biomass. Cytotoxicity and antimicrobial activity were evaluated by comparing the Ca/Mg/Zn-enriched biomass before γ-PGA functionalization with the corresponding Ca/Mg/Zn-enriched biomass after γ-PGA coating.

#### 3.2.2. γ-PGA Synthesis

Purified γ-polyglutamic acid (γ-PGA) was obtained commercially from Peptechlab Ltd. The polymer is biosynthesized and purified by the manufacturer utilizing their proprietary *Bacillus licheniformis* strain and scalable fermentation technology. The commercially sourced γ-PGA was used as received for the preparation of the immersion solutions, ensuring a standardized, highly reproducible biopolymer profile for the fungal biomass modification. The effectiveness of this system arises from the balanced provision of nutrients and environmental conditions that favor both cellular growth and metabolite production.

In particular, glucose ensures a sufficient energy supply, while glutamate availability directly drives polymer synthesis. Yeast extract contributes essential micronutrients, and the mineral fraction stabilizes enzymatic processes. Slightly acidic conditions further enhance the activity of enzymes involved in γ-PGA biosynthesis.

#### 3.2.3. γ-PGA Mycelium Immersion

The immersion of fungal biomass in γ-polyglutamic acid (γ-PGA) solutions was performed to enable surface modification and potential incorporation of the biopolymer into the mycelial structure. Prior to modification, the obtained biomass was thoroughly washed with 50 mL of distilled water three times to remove residual culture medium components, followed by freeze-drying and standardization based on dry mass.

For each experiment, 50 mg of lyophilized mycelium was weighed and immersed in 2 mL of γ-PGA solution (concentration: 150 g L^−1^ adjusted to pH 7). The immersion process was conducted at room temperature (22 ± 2 °C) for 24 h under continuous gentle agitation at 100 rpm to ensure uniform exposure, physical adsorption, and impregnation of the biopolymer into the mycelial matrix. Because no cross-linking agents were used, this process represents non-covalent physical coating rather than chemical coupling.

After immersion, the samples were dried in a vacuum desiccator for 48 h until a constant weight was achieved. The final mass of the dried γ-PGA-coated material was approximately 24.5 mg, corresponding to an estimated polymer loading efficiency of 10%.

### 3.3. Method Analysis

#### 3.3.1. Relative Dry Biomass Index

After 21 days of cultivation, the biomass was separated from the liquid medium via filtration. The control biomass (non-enriched, pure biomass) and biomass enriched with Ca, Mg, and Zn were lyophilized and weighed using an analytical balance with a precision of 0.001 g. The biomass yield was expressed as a percentage relative to the control, according to the following formula:

$$\mathrm{Percentage}\;\mathrm{relative}\;\mathrm{to}\;\mathrm{control}\;=\;\frac{x}{control}\;\times \;100\%,$$

x—dry biomass enriched with Ca, Mg or Ca.

#### 3.3.2. Metal Analysis

Freeze-dried mushroom biomass was pulverized and weighed (0.2 g) into Teflon vessels and subjected to microwave-assisted wet digestion. The samples were digested using a Multiwave 3000 system (Anton Paar, Buchs, Switzerland) with a reagent mixture consisting of 6 mL of concentrated nitric acid (65%, HNO_3_, Suprapure^®^, Merck, Darmstadt, Germany) and 2 mL of hydrogen peroxide (30%, H_2_O_2_, Merck, Darmstadt, Germany). After digestion, the solutions were diluted to 10 mL with quadruple-distilled water. Calcium, magnesium, and zinc levels were quantified using flame atomic absorption spectrometry (iCE3500, Thermo Scientific, Cambridge, Cambridgeshire, UK). All samples were prepared in triplicate, with three replicate measurements per sample.

#### 3.3.3. Microstructure Analysis

The morphology of *Pleurotus ostreatus* (Po) and *Pleurotus djamor* (Pdj) biomass, including unmodified samples and samples functionalized with poly(γ-glutamic acid) (γ-PGA), was examined by scanning electron microscopy (SEM). SEM observations were performed using a Hitachi SU-70 Schottky field-emission scanning electron microscope (FE-SEM; Hitachi High-Technologies, Hitachinaka, Japan) operated at an accelerating voltage of 30 kV.

Prior to imaging, the samples were mounted on copper grids and examined at variable magnifications. For each material variant, three independently prepared samples were analyzed. Several areas of each sample were observed, and representative micrographs were selected for qualitative comparison of the surface morphology and structural organization of the fungal biomass. Representative SEM images are presented in Figure 3.

#### 3.3.4. Analysis of the Surface and Grain Size of the Material

Nitrogen adsorption/desorption measurements were performed at liquid-nitrogen temperature (77 K) to determine the specific surface area of the biomass. For each measurement, 0.3 g of homogenized, freeze-dried sample was used. Prior to analysis, the samples were degassed under vacuum at 150 °C for 24 h. The measurements were conducted using an ASAP 2010 instrument (Micromeritics Instrument Corporation, Norcross, GA, USA). The specific surface area was calculated using the Brunauer–Emmett–Teller (BET) method from the adsorption branch of the nitrogen isotherm in the relative-pressure range (P/Po range) of 0.05–0.30.

Particle-size distribution was determined by wet laser-diffraction analysis using a Mastersizer 2000 particle-size analyser equipped with a Hydro S wet-dispersion unit (Malvern Instruments, Malvern, UK). The powdered samples were dispersed in an aqueous medium containing a fluidizing agent and subjected to high-energy ultrasonication to reduce agglomeration before measurement. The suspension was analyzed using the Hydro S wet-dispersion unit over the measurement range of 0.01–2000 µm. Particle-size results are expressed as volume-based percentile diameters D10, D50, and D90, where Dx represents the particle diameter below which x% of the cumulative particle-volume distribution is found.

#### 3.3.5. Surface Wettability Analysis

To evaluate the surface wettability, contact angle measurements were conducted on hydrogel samples containing fungal biomass. Polar and non-polar test liquids (diiodomethane, deionized water, oil, and cattle serum) were applied as 2 μL droplets to the material surface. The angle formed at the droplet–surface interface was recorded using a KRÜSS DSA 10 analyzer (KRÜSS GmbH, Hamburg, Germany).

#### 3.3.6. XPS Analysis

XPS analyses were performed using a PHI 5000 VersaProbe II Scanning XPS Microprobe (ULVAC-PHI, Chigasaki, Japan) equipped with a monochromatic Al Kα X-ray source (photon energy 1486.6 eV). The X-ray beam was focused to a 100 µm spot and raster-scanned over an area of 400 × 400 µm on the sample surface. All measurements were performed at a photoelectron take-off angle of 45° under ultra-high-vacuum conditions (pressure in the analysis chamber < 2 × 10^−9^ mbar).

#### 3.3.7. Thermal Analysis

The thermal stability (TG) of the tested biomaterials was examined using thermogravimetric analysis (TG). Approximately 5 mg of homogenized and finely ground lyophilized biomass was placed in a platinum crucible and heated from 40 °C to 600 °C under a nitrogen flow, maintaining a constant heating rate of 10 °C/min. TG measurements were performed with a Netzsch TG 309 Libra instrument (NETZSCH, Selb, Germany).

Complementary calorimetric measurements were performed using a Mettler Toledo DSC 3 system (Mettler-Toledo GmbH, Greifensee, Switzerland) equipped with an intracooler unit. Biomass portions of comparable mass (approximately 5 mg) were sealed in aluminum pans with perforated lids to allow the escape of volatile components during heating. The samples were analyzed under nitrogen (30 mL/min) at a heating rate of 10 K/min across the temperature range of 25–185 °C. The resulting DSC profiles were processed using Mettler Toledo STAR^®^ software (ver.16.5), enabling the identification and quantification of thermal transitions characteristic of the biomaterials.

#### 3.3.8. Microbiological Analysis

The antimicrobial properties of the tested materials were evaluated using the disk diffusion assay. The antimicrobial activity was evaluated against standard internationally recognized reference bacterial strains obtained from the American Type Culture Collection (ATCC, Manassas, VA, USA): *Enterococcus faecalis* (ATCC 29212), *Staphylococcus aureus* (ATCC 29213), and *Pseudomonas aeruginosa* (ATCC 27853). These strains were selected as representative models of key Gram-positive, Gram-negative, and enterococcal pathogens frequently isolated from infected acute, chronic, and surgical wounds. All strains were maintained and handled in strict accordance with EUCAST quality control guidelines. The bacterial suspensions were prepared in sterile 0.85% saline, adjusted to a 0.5 McFarland standard, and evenly distributed across Mueller–Hinton agar plates (bioMérieux, Warszawa, Poland) to obtain confluent growth. Sterile paper discs (6 mm diameter, Argenta, Poznań, Poland) were loaded with 20 µL of extract of the investigated formulations, including fungal biomass (*P. ostreatus*, *P. djamor*) in both untreated and γ-PGA-modified variants. Standard Gentamicin 10 µg discs and empty discs were applied simultaneously as positive and negative controls, respectively. All disk diffusion tests were performed in triplicate independent biological replicates. The discs were placed on the inoculated agar surfaces and incubated at 37 °C for 24 h. After incubation, the diameters of the clear inhibition zones surrounding each disc were measured in millimeters. All disk diffusion tests were performed in triplicate independent biological replicates. The interpretation of antimicrobial effectiveness followed the current EUCAST guidelines (version 15.0, 2025).

#### 3.3.9. Cytotoxicity Analysis

HaCaT keratinocytes were used as a representative epidermal model, as this human cell line is widely applied for assessing cytocompatibility and wound-relevant responses of dermal biomaterials. Human HaCaT cells (Sigma-Aldrich, Hamburg, Germany) were cultured in the recommended medium supplemented with 10% FBS and antibiotics at 37 °C in 5% CO_2_.

Pure fungal biomass (control) and biomass modified with the addition of γ-PGA were tested for cytotoxicity using the MTT assay according to ISO 10993-5:2009 guidelines. To prepare the primary extracts, the biomaterials were incubated in complete DMEM for 24 h at 37 °C to achieve a stock concentration of 50 mg/mL, corresponding to the ‘Bio50’ treatment group. The extracts were then sterile-filtered (0.22 µm) and subjected to a two-fold serial dilution in complete DMEM to generate the tested concentration gradient (Bio50, Bio25, Bio12.5, Bio6.25, Bio3.125, and Bio1.56). These varying concentrations were applied (100 µL/well) to HaCaT cells (5 × 10^3^ cells/well, ~80% confluency) and incubated for 24 h. Cells cultured in standard complete DMEM without any material extract served as the negative control (representing 100% viability), while cells treated with 10% DMSO served as the positive cytotoxic control. Following the exposure period, MTT (0.5 mg/mL) was added for 4 h, the formed formazan crystals were solubilized in DMSO, and absorbance was recorded at 570 nm with a 630 nm reference.

### 3.4. Reagents

#### 3.4.1. Liquid Culture Media

Calcium chloride (CaCl_2_), magnesium sulfate heptahydrate (MgSO_4_$\cdot 7H_{2}O)$ and zinc sulfate heptahydrate (ZnSO_4_$\cdot 7H_{2}O)$ used for medium supplementation were obtained from Sigma-Aldrich (Merck KGaA, Darmstadt, Germany, 2025).

#### 3.4.2. Mineralization of Freeze-Dried Biomass

Freeze-dried samples were subjected to microwave-assisted digestion using 65% nitric acid (HNO_3_; Suprapure^®^, Merck, Darmstadt, Germany) together with 30% hydrogen peroxide (H_2_O_2_, Suprapure^®^, Merck, Darmstadt, Germany). All reagents and dilutions were prepared using quadruple-distilled water with conductivity below 1 μS/cm, produced with an HLP5 purification system (Hydrolab, Straszyn, Poland).

#### 3.4.3. AAS Measurements

Certified 1 g/L stock standard solutions of calcium (II), magnesium (II), and zinc (II) were obtained from Sigma-Aldrich (Darmstadt, Germany). These standards were used to prepare calibration solutions for the quantitative determination of the respective elements using flame atomic absorption spectrometry (FAAS).

### 3.5. Statistical Analysis

Data processing and statistical analyses were conducted using the R programming environment (RStudio 2026.04.0+526). To evaluate the effects of different media additives and fungal species on the studied parameters (such as biomass growth, metal accumulation, cell viability, and antimicrobial activity), generalized linear mixed models (GLMMs) were employed. GLMMs were specifically chosen because they provide a robust framework for handling non-normally distributed, continuous biological data while simultaneously accounting for both fixed experimental factors and random sources of variance.

In the model specification, the fungal species (*Pleurotus ostreatus* and *Pleurotus djamor*), specific media treatments (control, independent additions of Ca, Mg, and Zn at 200 mg and 500 mg, and their multi-element mixture), and their two-way interactions were designated as fixed categorical factors. The fundamental biological unit for all statistical analyses was defined as the independent in vitro cultivation flask (representing a distinct batch of biomass). To strictly avoid pseudo-replication, any technical or instrumental replicates—such as the three sequential instrument readings per sample in the metal analysis or multiple technical wells in the MTT assay—were first averaged to generate a single aggregate value per independent biological unit. Consequently, the sample size in our models reflects only true biological replicates. To account for the natural variance and baseline differences across independent experimental runs, the biological batch (‘Repetition’) was incorporated into the models as a random intercept factor. To ensure the highest model accuracy, multiple probability distributions and linking functions were systematically evaluated for the response variables. The tested configurations included Gaussian distributions (with identity and log link functions) and gamma distributions (with inverse and log link functions). The final best-fitting model for each dataset was selected based on the Akaike Information Criterion (AIC). The model demonstrating the lowest AIC value was identified as the optimal fit using the model.sel function from the MuMIn package.

All mixed modeling was performed using the lme4 package. Model tidying and summary extractions of the fixed effects (including coefficients, standard errors, and *p*-values) were facilitated by the broom.mixed and dplyr packages, and the results were subsequently exported using writexl. Finally, data visualizations—specifically, violin plots overlaid with jittered data points to illustrate both the data distribution and individual observations—were generated using the ggplot2 package.

## 4. Conclusions

This study demonstrates the successful development and modification of a novel biomaterial based on *Pleurotus* spp. biomass enriched with vital trace elements (Ca, Mg, Zn) and coated with γ-polyglutamic acid (γ-PGA). Our in vitro experimental findings highlight a fundamental biological trade-off that is highly relevant to the conceptual design of biomaterials for chronic wound management. Specifically, while the raw fungal matrix—particularly *P. ostreatus*—possesses natural in vitro antimicrobial properties against key pathogens such as *S. aureus* and *P. aeruginosa*, it simultaneously exhibits dose-dependent cytotoxicity toward human keratinocytes (HaCaT) at high concentrations. The incorporation of γ-PGA introduces a crucial cytoprotective effect, successfully rescuing keratinocyte viability at these high biomass concentrations and neutralizing the inherent acute toxicity of the fungal matrix.

Although the addition of γ-PGA partially attenuates the macroscopic bactericidal efficacy of the raw fungal biomass—evidenced by reduced inhibition zones for *S. aureus* and *P. aeruginosa* and the complete resistance of *E. faecalis*—this represents a justified compromise in biomaterial design, prioritizing host–cell viability over maximum pathogen eradication. By balancing basic bacteriostatic properties with the preservation of delicate human cells, the γ-PGA-modified *Pleurotus* matrix emerges as a highly promising, biocompatible active ingredient for advanced biomaterials.

It must be emphasized that these findings are strictly in vitro and characterize the functionalized biomass itself rather than a finalized medical device. To translate this bioactive matrix into a clinical application, further investigation is strictly required. Future studies must focus on the actual fabrication of complete wound-dressing systems (e.g., hydrogels) incorporating this fungal matrix. This must include the precise quantification of γ-PGA retention following washing cycles and the spatial mapping of polymer distribution within the bulk matrix. Furthermore, to fully decouple the therapeutic contributions of the raw matrix, the polymer, and the biofortification process, future research must systematically compare the biological activities of unmodified, individually metal-enriched, and γ-PGA-coated materials. This must be paired with experiments evaluating the quantitative release behavior of the active components (specifically zinc, calcium, magnesium, and antimicrobial metabolites) under simulated physiological conditions to clarify the final safety and efficacy profiles of the resulting biomaterials. These mechanistic material evaluations must be paired with advanced, quantitative microbiological assessments—specifically viable bacterial counts, dynamic time–kill kinetic assays, and antibiofilm activity evaluations—to accurately decouple diffusion limitations from true bactericidal efficacy. Combined with comprehensive in vivo safety and wound-healing studies in appropriate animal models, these steps are strictly required to translate this bioactive matrix into clinical application.

### Limitations and Future Perspectives

It should be noted that the present study utilized standard ATCC reference strains (*S. aureus* ATCC 29213, *P. aeruginosa* ATCC 27853, and *E. faecalis* ATCC 29212) to establish a standardized, highly reproducible baseline for antimicrobial screening in accordance with international EUCAST standards. While these quality-control strains are representative of major bacterial species causing wound infections, antimicrobial susceptibility can vary among different clinical isolates and multidrug-resistant (MDR) strains. Therefore, while these baseline findings demonstrate clear proof-of-concept antimicrobial efficacy, future translational studies will expand this evaluation to clinically derived isolates to confirm broader therapeutic applicability.

## Figures and Tables

**Table 1:** Surface chemical composition of *Pleurotus djamor* (Pdj) and *Pleurotus ostreatus* (Po) biomass cultivated with and without γ-polyglutamic acid (γ-PGA), determined by XPS high-resolution spectral deconvolution (at.%).

Chemical Bonds/Groups/Compounds	C–C/ C–H	C–O	C=O	O–C=O	N–H/ C–N	Protonated N	O=C	O–C/ OH	H_2_O/ Adsorbed Species	P	S	Cl
Pdj	35.6	22.8	8.5	2.6	3.6	0.7	5.7	17.3	2.8	0.4	0.0	0.1
Po	29.3	26.2	8.1	2.3	1.7	0.7	3.7	24.5	2.7	0.3	0.2	0.3
Pdj-PGA	36.8	20.9	8.4	2.5	2.7	2.1	7.2	17.2	1.7	0.4	0.1	0.2
Po-PGA	31.0	24.7	8.6	2.5	1.8	0.8	3.2	24.8	1.7	0.4	0.1	0.3

Values represent the atomic concentrations (at.%) of fitted XPS components; values may not sum to exactly 100% because of rounding.

## Data Availability

The original contributions presented in this study are included in the article. Further inquiries can be directed to the corresponding author.
